# Effectiveness of tuberculosis preventive treatment on disease incidence among people living with HIV/AIDS: A systematic review and meta-analysis

**DOI:** 10.1371/journal.pone.0330208

**Published:** 2025-08-26

**Authors:** José Nildo de Barros Silva Júnior, Gilberto da Cruz Leal, Quézia Rosa Ferreira, Licia Kellen de Almeida Andrade, Jaqueline Garcia de Almeida Ballestero, Victor Santana Santos, Júlia M.a Pescarini, Anete Trajman, Denise Arakaki-Sanchez, Patricia Bartholomay, Rubia Laine de Paula Andrade, Daniele Pelissari, Pedro Fredemir Palha

**Affiliations:** 1 Ribeirão Preto College of Nursing, University of São Paulo, Ribeirão Preto, SP, Brazil; 2 Institute of Scientific and Technological Communication and Information in Health, Oswaldo Cruz Foundation, Rio de Janeiro, RJ, Brazil; 3 Department of Medicine, Federal University of Sergipe, Lagarto, SE, Brazil; 4 London School of Hygiene & Tropical Medicine, London, GLA, United Kingdom; 5 Research Institute, McGill University Health Center, Montréal, QC, Canada; 6 Faculty of Medicine, Federal University of Rio de Janeiro, Rio de Janeiro, Brazil; 7 Ministry of Health, National Tuberculosis Programme, Brasilia, DF, Brazil; 8 Ministry of Health, National Center for Epidemiological Intelligence and Genomic Surveillance, Brasilia, DF, Brazil; Stop TB Partnership, UNOPS, SWITZERLAND

## Abstract

**Background:**

Clinical trials have shown the protective efficacy of tuberculosis preventive treatment (TPT) for averting disease and death from tuberculosis among people living with HIV/AIDS (PLHIV). TPT has been recommended for PLHIV since the 1980s. However, tuberculosis is still the first cause of death in PLHIV.

**Objective:**

We aimed to summarize the evidence related to the real-world effectiveness of TPT on tuberculosis incidence among PLHIV.

**Method:**

This is a systematic review and meta-analysis of observational cohort studies. The search was carried out in PubMed (via MEDLINE), Embase, LILACS, Scopus and Web of Science databases. Free and controlled vocabulary was used for the searches, with no restrictions on language or publication period. Studies reporting hazard ratios (HR) for tuberculosis incidence among PLHIV who received TPT were pooled using random-effects meta-analysis models. Meta-regression was performed to assess whether study-level characteristics accounted for heterogeneity, as evaluated by Cochran’s I² statistic. Study quality was appraised using the Newcastle-Ottawa Scale. This study was registered with PROSPERO (CRD42024586273).

**Results:**

Among 8,330 screened studies, 34 were included, with nine contributing to the meta-analysis. TPT was associated with a 63% reduction in tuberculosis incidence risk (HR = 0.37, 95% CI: 0.28–0.48; I² = 43%). Children exhibited consistent stronger protection (82% risk reduction, HR = 0.18, 0.09–0.37; I² = 0%) than adults (56% reduction, HR = 0.44, 0.37–0.53; I² = 21%).

**Conclusion:**

In real world conditions, TPT significantly and substantially reduces tuberculosis incidence in PLHIV, with consistent evidence of stronger protective effects in children. Despite some heterogeneity among adult studies, the pooled evidence confirms the protective effectiveness previously observed in clinical trials. These findings reinforce the global recommendation for broad implementation of TPT among PLHIV.

## Introduction

Acquired immunodeficiency syndrome (AIDS) and tuberculosis are two of the leading infectious causes of global morbidity and mortality. In 2023, approximately 10.8 million people fell ill with tuberculosis, resulting in 1.25 million deaths, including 161,000 among people living with the human immunodeficiency virus (PLHIV) [1]. That same year, tuberculosis regained its position as the leading cause of death from a single infectious agent in the world, after three years being overtaken by the coronavirus disease (COVID-19), and was responsible for almost twice as many deaths as HIV/AIDS [1].

The combination of tuberculosis and HIV/AIDS can be lethal due to the synergistic interaction between the two diseases, which amplifies mutual severity. HIV-induced immunosuppression compromises the cellular immune response, increasing susceptibility to primary infection and reactivation of tuberculosis infection (TBI) [2]. This interaction significantly reduces the survival of PLHIV, especially those with low CD4 T-lymphocyte counts and in settings with a high burden of infectious diseases [3,4].

During the 1980s, the World Health Organization (WHO) and other institutions recommended using isoniazid for preventing tuberculosis, especially in areas with a high prevalence of the disease and population at high risk to develop tuberculosis disease [5]. WHO currently recommends shorter rifampicin-based regimens for tuberculosis preventive treatment (TPT), but isoniazid remains an option [6] From the perspective of the Sustainable Development Goals (SDG), in 2015, the WHO declared that achieving the goal of eliminating tuberculosis would only be possible with the broad expansion of TPT alongside continuing to detect and treat persons with tuberculosis [5]. However, the development of tuberculosis disease among PLHIV remains a global concern [7].

HIV-induced immunosuppression increases the risk of reactivation, even after preventive treatment for tuberculosis has been administered. It is estimated that the risk of progression from TBI to tuberculosis disease among PLHIV is 14–18 times higher than in the general population [1]. Non-adherence to antiretroviral therapy (ART) and the presence of comorbidities or coinfections can further compromise the effectiveness of TPT [7,8].

Understanding the impact of TPT on tuberculosis incidence among PLHIV is crucial not only to addressing the gaps in the global response to tuberculosis-HIV co-infection, but also to advancing the goal of eliminating tuberculosis as a public health problem.

Systematic reviews that consolidate global evidence provide critical support for developing more targeted policies and interventions for tuberculosis control [8]. While previous reviews on TPT among PLHIV have focused mainly on clinical trials [9–11], these may not reflect long-term outcomes or real-world implementation challenges. Moreover, some reviews did not specifically assess tuberculosis incidence among PLHIV who received TPT, or were limited to evidence from a single country, such as the cohort-based review by Geremew et al. [12], whereas the present study adopts a global perspective.

To evaluate the sustained benefits of TPT, this systematic review aimed to summarize the evidence related to the effectiveness of TPT on the incidence of tuberculosis among PLHIV in cohort studies.

## Methods

### Search strategy and selection criteria

This is a systematic review and meta-analysis of cohort studies whose protocol was registered in the PROSPERO (CRD42024586273), based on the guidelines included in the Joanna Briggs Institute Manual for Evidence Synthesis [13], the Preferred Reporting Items for Systematic Review and Meta-Analysis (PRISMA) [14] and Meta-Analyses Of Observational Studies in Epidemiology (MOOSE) [15]. The PRISMA Checklist is available at S1 Table.

The research question, the objective of the study and the descriptors use the mnemonic combination PECO, where P (Population) is PLHIV; E (Exposure) is TPT; C (Control) is PLHIV not undergoing tuberculosis preventive treatment and O (Outcome) is the incidence of tuberculosis. In other words, the guiding question of this review was: What is the summarized evidence related to the effectiveness of TPT on the incidence of tuberculosis among PLHIV?

Different combinations of search terms related to HIV, TPT and tuberculosis disease incidence were used. The Boolean operator ‘OR’ was used within each group and the Boolean operator ‘AND’ among the groups. Table 1 displays the descriptors used according to the application of their respective acronym.

**Table 1:** Descriptors found through automatic and manual searches.

Application of the acronym		Initial descriptors	Descriptors found
P	People living with HIV	HIV	HIV OR “HIV Infection” OR “HIV Infections” OR “Human Immunodeficiency Virus” OR “Human Immunodeficiency Virus” OR “Human Immuno-deficiency Virus” OR “Human Immune-deficiency Virus” OR “Human Immuno deficiency Virus” OR “Human Immune deficiency Virus” OR AIDS OR “Acquired Immunodeficiency Syndrome” OR “Acquired Immunodeficiency Syndrome” OR “Acquired immuno-deficiency syndrome” OR “Acquired immune-deficiency syndrome” OR “Acquired Immune Deficiency Syndrome” OR “Acquired Immuno Deficiency Syndrome” OR “HIV infection” OR “HIV infections” OR “Human Immunodeficiency Virus” OR “Acquired Immunodeficiency Virus” OR “Acquired Immunodeficiency Syndrome” OR HIV OR “Infección por VIH” OR “Infecciones por VIH” OR “Virus de inmunodeficiencia humana” OR “Virus de inmunodeficiencia adquirida” OR SIDA OR “Síndrome de inmunodeficiencia adquirida”
E	Tuberculosis Preventive Treatment	Tuberculosis Preventive Treatment	“Tuberculosis Preventive Treatment” OR “tb Preventive Treatment” OR “Treatment of Tuberculosis Infection” OR “Treatment of tb Infection” OR “Latent *M. tuberculosis* Infection Treatment” OR “Latent *Mycobacterium tuberculosis* Infection Treatment” OR “Treatment of latent tuberculosis infection” OR “Treatment of latent tb infection” OR “tuberculosis preventive therapy” OR “tb preventive therapy” OR “Latent Tuberculosis treatment” OR “Latent tb treatment” OR “Latent Tuberculosis therapy” OR “Latent tb therapy” OR “tuberculosis infection treatment” OR “tb infection treatment” OR “tuberculosis infection therapy” OR “tb infection therapy” OR “isoniazid preventive therapy” OR “isoniazid preventive treatment” OR ipt OR “Tratamento preventivo de tuberculose” OR “Tratamento preventivo de tb” OR “Tratamento de infecção por tuberculose” OR “Tratamento de infecção por tb” OR “Tratamento de infecção latente por M. tuberculosis” OR “Treatment of latent Mycobacterium tuberculosis infection” OR “Treatment of latent tuberculosis infection” OR “Treatment of latent tuberculosis infection” OR “preventive tuberculosis therapy” OR “preventive tb therapy” OR “Treatment of latent tuberculosis” OR “Treatment of latent tb” OR “Therapy of latent tuberculosis” OR “Therapy of latent tb” OR “treatment of tuberculosis infection” OR “treatment for tuberculosis infection” OR “therapy for tuberculosis infection” OR “therapy for tb infection” OR “preventive therapy with isoniazid” OR “preventive treatment with isoniazid” OR “Tratamiento preventivo de la tuberculosis” OR “Tratamiento de la infección por tuberculosis” OR “Tratamiento de la infección latente por M. tuberculosis” OR “Tratamiento de la infección por Mycobacterium tuberculosis latente” OR “Tratamiento de la infección por tuberculosis latente” OR “terapia preventiva de la tuberculosis” OR “Tratamiento de la tuberculosis latente” OR “Terapia de la tuberculosis latente” OR “tratamiento de la infección de tuberculosis” OR “tratamiento de la infección por tuberculosis” OR “terapia de la infección por tuberculosis” OR “terapia de la infección de tuberculosis” OR “terapia preventiva con isoniazida” OR “tratamiento preventivo con isoniazida”
C	People living with HIV not undergoing tuberculosis preventive treatment	^*^	
O	Incidence of tuberculosis disease	Incidence of tuberculosis disease	“tb disease incidence” OR “tuberculosis disease incidence” OR “tb disease development” OR “tuberculosis disease development” OR “develop tb” OR “develop tuberculosis” OR “developing tb” OR “developing tuberculosis” OR “developed tb” OR “developed tuberculosis” OR “tb incidence” OR “tb incident cases” OR “incident tb” OR “incident tuberculosis” OR “tb disease development” OR “incidence of tuberculosis disease” OR “development of tuberculosis disease” OR “developing tuberculosis” OR “developing tb” OR “development of tuberculosis” OR “development of tb” OR “developed tuberculosis” OR “incidence of tuberculosis” OR “incident cases of tuberculosis” OR “incident tuberculosis” OR “incident tuberculosis” OR “development of tuberculosis disease” OR “development of TB disease” OR “progresión de la tuberculosis” OR “desarrollo de la tuberculosis” OR “desarrollar tuberculosis” OR “incidencia de tuberculosis” OR “casos incidentes de tuberculosis” OR “tuberculosis incidente” OR “desarrollo de la tuberculosis enfermedad”

* The descriptors related to the component C (Control) are covered by the components P (Population) and E (Exposure).

Source: the authors.

The search was carried out on September 1, 2024 in the following databases: PubMed via Medical Literature Analysis and Retrieval System Online (MEDLINE), Excerpta Medica Database (EMBASE), Latin American and Caribbean Literature in Health Sciences (LILACS), SciVerse Scopus (SCOPUS) and Web of Science, accessed via the journal portal of the Coordination for the Improvement of Higher Education Personnel (CAPES). These databases were selected due to their extensive coverage and relevance to the subject under investigation, to enhance the representativeness and validity of the evidence incorporated into this review. The detailed search strategies tailored to each of the defined databases are provided in the Supporting Information (S2 Table).

### Inclusion and exclusion criteria

Eligible studies included prospective or retrospective cohorts of PLHIV who received or not TPT and were monitored for subsequent tuberculosis incidence, irrespective of language and publication year.

We excluded studies based on the following criteria (details on the reason for exclusion of each article considered for full reading are provided in S3 Table): other types of study (reviews, case reports, clinical trials, cross-sectional studies, or other non-cohort designs); conference abstracts (conference abstracts were excluded due to limited methodological detail and unavailability of full data); subject-obstacle (studies that did not clearly involve PLHIV, or that did not assess tuberculosis incidence as an outcome after TPT initiation); and gray literature (documents from the gray literature, including reports, dissertations, and other non–peer-reviewed sources).

Duplicates were removed and two authors (JNBSJ and GCL) independently selected the studies by reading the titles and abstracts of the studies identified, using the Rayyan QCRI [16].

If there were disagreements in the evaluations of the articles, a third author (QRF) was consulted. The final sample was selected by reading all the material. In cases where multiple publications of a study or cohort were identified, the publications with the most recent data were included. Deepl platform was used to translate abstracts from languages other than Portuguese and English, to guarantee the eligibility of the titles selected.

### Data extraction

Using a standardized data extraction form, four authors (JNBSJ, GCL, QRF and LKAA) extracted information from the studies (first author, year of publication, country of study, period of analysis, study setting, objective, study population, eligibility criteria for TPT and effectiveness of TPT on the incidence to active tuberculosis).

The information extracted from the studies was organized in a Google Sheets spreadsheet, ensuring online accessibility and allowing collaboration among the authors in real time. All steps and information extracted from the studies underwent double validation (JNBSJ and GCL).

### Meta-analysis and statistical analysis

Studies reporting hazard ratios (HRs) for tuberculosis incidence among PLHIV who received TPT were pooled using random-effects meta-analysis models, prioritizing the most adjusted estimates when available. Effect measures were log-transformed (logHR) to ensure distributional symmetry and to allow proper combination of effect sizes. The meta-analysis was conducted using the restricted maximum likelihood (REML) method to estimate between-study heterogeneity (τ²). This model was selected due to the expected clinical and methodological variability among the study populations. The pooled HR and its 95% confidence interval (CI) were calculated, and heterogeneity was assessed using the I² statistic.

Studies were stratified by age group (adults and children) to explore potential sources of heterogeneity, as per protocol. Subgroup differences were tested using the Q-test for heterogeneity between groups. In addition, meta-regression analyses were performed to formally assess whether the effect of TPT on tuberculosis incidence varied by age group and ART use. The age group variable (children vs. adults) showed a statistically significant interaction with the estimated effect size.

A forest plot was constructed to visualize the pooled effect, and a funnel plot was used to assess potential publication bias through visual inspection. Due to the inclusion of subgroups in the meta-analysis model, Egger’s regression test was not applied, as it is not appropriate for models including subgroups. All statistical analyses were conducted using R (version 4.5.0) with the *meta* and *metafor* packages.

### Assessment of the methodological quality of the studies included in the review

To evaluate the methodological quality of the included studies, we used the Newcastle-Ottawa Scale (NOS) [17]. This tool, commonly applied in cohort studies, assesses three domains: selection of the exposed and non-exposed cohorts (maximum of four stars), comparability of the cohorts (maximum of two stars), and assessment of outcomes (maximum of three stars), with one star awarded for each item adequately fulfilled. Two authors (QRF and LKAA) independently conducted the quality assessment, and any discrepancies were resolved through consultation with a third author (JNBSJ). No studies were excluded based on the methodological quality assessment. Detailed information regarding this evaluation is available in the Supporting Information (S4 Table), presenting the full results.

### Ethical aspects

Ethical approval was not required as this study used publicly available data.

## Results

**Fig 1:**
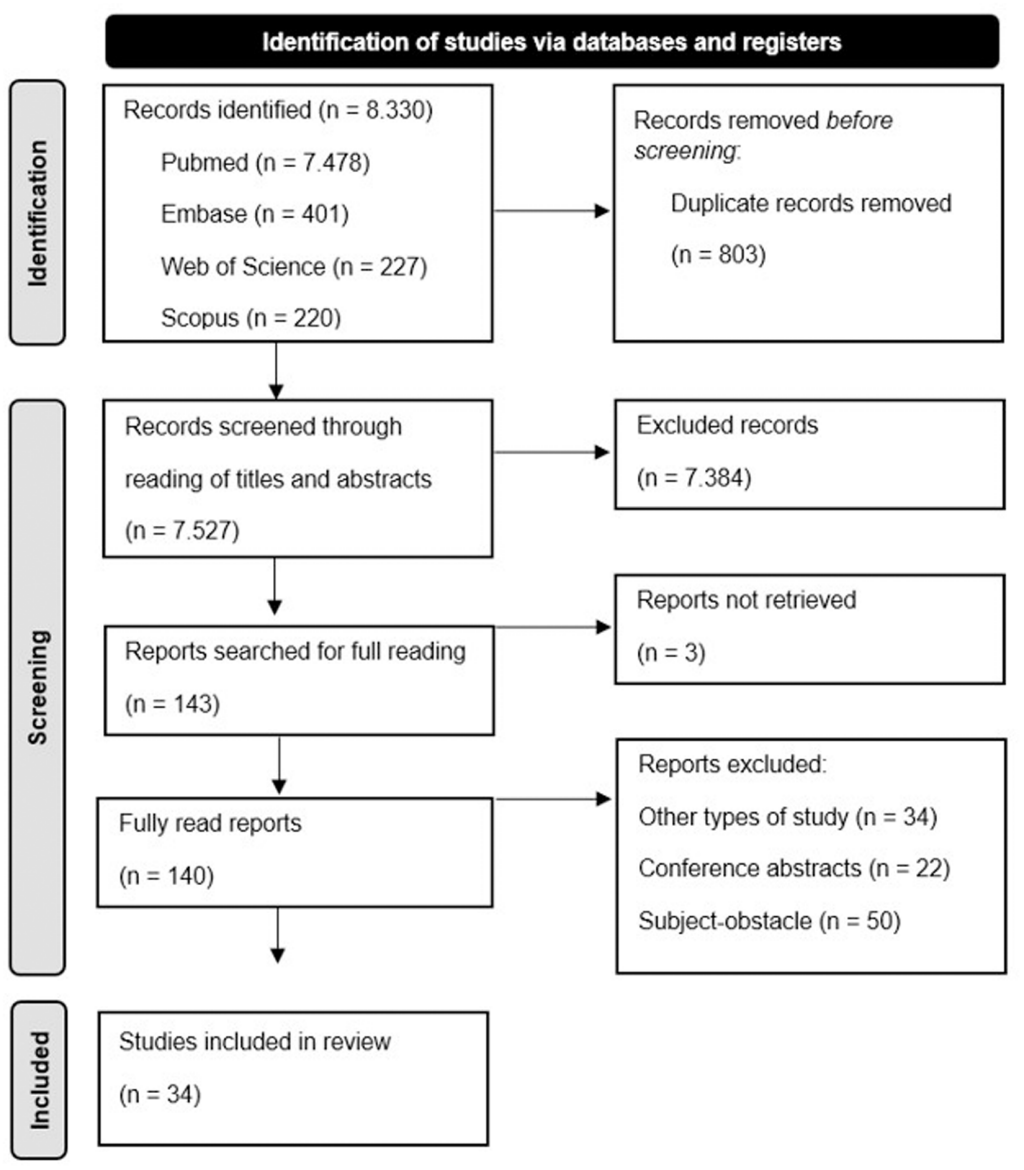
PRISMA flowchart. Source: created from the model by Page *et al*. [14].

The search across the selected databases yielded a total of 8,330 documents. After excluding duplicates, we included 34 [18–51] studies published between 1999 and 2024. The flowchart of the steps taken to include the studies can be seen in Fig 1. Table 2 presents the information and synthesis of the main results of the studies selected for this systematic review.

**Table 2:** Information and synthesis of the main results of the studies selected for the systematic review.

Authors/ Year/ Country	Period of analysis/ Study scenario	Objective	Study population/ Eligibility criteria for TPT	Effectiveness of TPT on the incidence to active TB
Ajema *et al*, 2024 [18] Ethiopia	2023 Health facilities in the city of Hawassa, Ethiopia.	To assess the incidence and predictors of time to development of TB among PLHIV attending follow- up care at health facilities	- 393 PLHIV were followed up for a total of 862.68 PY - PLHIV ≥15 years on ART for ≥6 months - No active TB at TPT initiation	- TB incidence: • With completed TPT: 1.05/100 PY • Without completed TPT: 44.73/100 PY - Risk reduction: 6x higher TB risk with incomplete TPT (aHR = 6.2; 95% CI: 2.34–16.34)
Mulatu *et al*, 2023 [19] Brazil	2016- 2022 5 public hospitals and 3 health centers offer ART services.	To evaluate incidence of TB in PLHIV and factors associated among those using ART	- 422 PLHIV - Adult PLHIV on ART - No active TB at TPT initiation - No contraindications to isoniazid	- TB incidence: • With TPT: 1.7/100 PY - aHR = 0.49 (95% CI: 0.25–0.96) • Without TPT: 7.1/100 PY
Nyangu *et al.*, 2022 [20] Zambia	2016- 2019 608 public health units in the provinces of Lusaka, West, East and South.	To determine the incidence and predictors of TB among PLHIV who were on TPT in Zambia.	- 48,581 PLHIV - PLHIV without TB symptoms (cough, weight loss, fever, night sweats) and without contraindications (hepatitis, peripheral neuropathy, isoniazid hypersensitivity, etc.). - Active TB ruled out before TPT initiation.	- TB incidence: • During TPT (breakthrough TB): 0.3% (130/48,581). - Risk reduction: Significant risk reduction after the first month. − 79% of cases occurred during the first month of TPT, suggesting missed diagnoses or TB- associated immune reconstitution inflammatory syndrome.
Onyango *et al.*, 2022 [21] Kenya	2015- 2019 8 HIV clinics in Kisumu County.	To evaluate the effectiveness of TPT in children living with HIV..	- 856 PLHIV - PLHIV aged ≥12 months and <15 years. - No symptoms of active TB or active TB ruled out after evaluation.	- TB incidence: • With completed TPT: 2.1/1000 PY • Without TPT (not initiated): 8.1/1000 PY • TPT initiated but not completed: 9.2/1000 PY
Kazibwe *et al*., 2022 [22] Uganda	2016- 2021 11 centers of AIDS Support Organization.	To determine the incidence, associated factors and median time to diagnosis of TB among PLHIV on ART who have started preventive therapy with isoniazid.	- Records of 2,634 PLHIV on ART - No active TB (excluded through clinical screening using WHO ICF form)	- TB incidence: • With completed TPT: ~ 0.019/100 PY • With interrupted TPT: 24.02/1,000 PY - TPT interruption associated with 25x higher TB risk (aHR: 25.96; 95% CI: 4.12–169.48)
Geremew *et al.*, 2022 [23] Ethiopia	2016- 2019 Specialized hospital University of Gondar.	To evaluate the incidence and predictors of TB among HIV- positive adult patients on ART.	- 368 PLHIV - Adults with HIV (≥18 years) on ART - No diagnosis of active TB at study initiation	- TB incidence: • With TPT: 8.4% (10/119) • Without TPT: 21.3% (46/216) - Risk reduction: 2.8x (AOR: 2.8; 95% CI: 1.3–5.9)
Russom *et al*., 2022 [24] South Africa	2016- 2018 All the HIV clinics of the national and regional referral hospitals (including private) in Eritrea.	To evaluate the impact of TPT on reducing the incidence of TB and the duration of its protection in PLHIV. And to measure the effect of TPT on reducing all- cause mortality.	− 6,803 PLHIV - PLHIV on ART - No active TB at TPT initiation - At least 1 year of follow- up recorded	- TB incidence: • With TPT: 1.7/1000 PY • Without TPT: 10/1000 PY - Risk reduction: 74% (aHR: 0.26; 95% CI: 0.17–0.40) - Protection duration: Declined after 6 months post- TPT completion
Maokola *et al*, 2021 [25] Tanzania	2012- 2016 315 HIV clinics.	To evaluate the effectiveness of TPT in routine clinical settings, comparing the incidence of TB between the TPT and non- TPT groups.	- Records of 161.417 PLHIV - PLHIV (adults and children) without confirmed active TB - Implemented in HIV clinics with available IPT	- TB incidence: • With TPT: 10.49/1000 PY • Without TPT: 12.00/1000 PY - Risk reduction: 52% (aHR = 0.48; 95% CI: 0.40–0.58)
Mandalakas *et al.*, 2021 [26] South Africa	2007- 2012 Communities served by the local public health program (community areas in Durban, KwaZulu- Natal).	To evaluate the effectiveness of TB prevention interventions for children in a community in South Africa.	- 966 children - Children <5 years or living with HIV - Recent TB exposure or positive immunological test (M. tuberculosis)	- TB incidence: • With TPT: 2.2% (6/276) • Without TPT: 3.4% (21/617) - Risk reduction: 82% (adjusted OR: 0.18; 95% CI: 0.06–0.52)
Kebede *et al*., 2021 [27] Ethiopia	2016- 2019 2 general hospitals (Assosa and Pawe)	To evaluate the effect of TPT on the incidence of TB in HIV- positive children in northwest Ethiopia.	- Records of 421 HIV- positive children - HIV- positive children ≤15 years receiving HIV/AIDS care - Exclusion criteria: active TB at baseline or incomplete clinical data	- TB incidence: • With TPT: 1.14/100 PY • Without TPT: 11.5/100 PY - Risk reduction: 96.8% (aHR = 7.45; 95% CI: 2.96–18.74)
Souza *et al.*, 2021 [28] Brazil	2003- 2016 Evandro Chagas National Institute of Infectious Diseases.	To evaluate the incidence of TB and associated factors in PLHIV.	- 138 PLHIV - HIV- positive adults with tuberculin skin test (TST) ≥5 mm - Exclusion criteria: active TB at study enrollment	- TB incidence: • Completed TPT: 5% (6/119) - Probability of developing TB in 10 years after IPT: 5.4%
Beshaw, Balcha, Lakew, 2021 [29] Ethiopia	2008- 2015 Gondar and Azezo health centers in the city of Gondar.	To evaluate the effect of IPT and associated factors among HIV- infected individuals.	- Records of 450 PLHIV - PLHIV ≥18 years (adults) in pre- ART or ART care Exclusion criteria: - Active TB at TPT initiation - Contraindications to isoniazid	- TB incidence: • Completed TPT: 0.35/100 PY • Without TPT: 7.1/100 PY - Risk reduction: 92% (aHR = 0.08; 95% CI: 0.02–0.37)
Padmapriya darsini *et al.*, 2020 [30] India	2013- 2016 Seven ART centers located in urban and suburban India.	To assess the feasibility of implementing TPT under field conditions and to evaluate its effectiveness in terms of compliance, treatment outcome, adverse event and post- TPT effect, when IPT was initiated at different CD4 cell counts, with or without ART, in real- life settings.	- 4,528 PLHIV - PLHIV ≥18 years - TB- asymptomatic (no cough, fever, night sweats, or weight loss) - No active TB or prior TB treatment - No contraindications (hepatopathy, alcoholism, etc.)	- TB incidence: • With TPT: 1.17/100 PY • Without TPT (pre- intervention): 2.42/100 PY • Post- TPT (6 months): 0.64/100 PY - Risk reduction: 52% (p < 0.001)
Aemro, Jember, Anlay, 2020 [31] Ethiopia	2014- 2018 Debre Markos Referral Hospital.	To evaluate the incidence and predictors of TB among adults on ART	- Records of 494 PLHIV - HIV+ adults on ART - Exclusion: active TB at the start of the study or TB status not recorded	- TB incidence: • With TPT: 1.23/100 PY (95%CI: 0.51–2.95) • Without TPT: 9.61/100 PY (95%CI: 7.41–12.45) - Risk reduction: 67% (aHR = 0.33; 95%CI: 0.12–0.85)
Atey *et al.*, 2020 [32] Ethiopia	2010- 2016 6 TARI clinics from selected hospitals in northern Ethiopia, Tigray Regional State.	To determine the effect of IPT on the incidence of TB, follow- up CD4 + T cells and the all- cause mortality rate.	- 1,863 PLHIV - Adult PLHIV on ART - No active TB at start of TPT - Exclusion of liver disease, epilepsy or alcohol dependence (AUDIT >8)	- TB incidence: • With TPT: 620/100,000 PY • Without TPT: 3,160/100,000 PY - Risk reduction: 80% (aHR = 0.339; 95%CI: 0.211–0.545)
Mengesha, Ahmed, 2020 [33] Ethiopia	2016- 2018 Dessie Referral Hospital.	To evaluate the outcome of IPT, including the prevalence of TB and other opportunistic infections	- Records of 220 PLHIV - Adult PLHIV on ART - No active TB at start of TPT - Exclusion of patients transferred or with incomplete records	- TB incidence: • With TPT: 4.09% (9/110) • Without TPT: 7.27% (16/110) • Difference not statistically significant (p = 0.137)
Yirdaw *et al.*, 2019 [34] Ethiopia	2005- 2014 11 hospitals in Addis Ababa.	To evaluate the magnitude and determinants of TB reactivation among PLHIV who received TPT.	- 4,484 PLHIV - PLHIV without a diagnosis of active TB (WHO symptom- based screening) - No contraindications for isoniazid	- Risk reduction with ART: - Adjusted HR: 0.08 (95%CI: 0.04–0.14) - Greater protection in patients with CD4 ≥ 350 cells/mm³ (HR: 0.12; 95%CI: 0.05–0.28) - TB incidence: • During TPT (breakthrough TB): 1106/100,000 PY • With completed TPT: 624/100,000 PY
Sabasaba et al., 2019 [35] Tanzania	2010- 2017 HIV clinics in Dar es Salaam.	To evaluate the effectiveness of TPT in reducing the incidence of TB among PLHIV.	- 68,378 PLHIV - PLHIV ≥15 years without signs or symptoms of active TB - Treated for TB for more than 2 years	- TB incidence: • With TPT: 1.54/100 PY • Without TPT: 2.94/100 PY - Risk reduction: TPT associated with 48% lower TB incidence (IRR = 0.52; 95%CI: 0.46–0.59)
Wong *et al.*, 2019 [36] Hong Kong	2002- 2017 HIV clinic with a case load exceeding 3,000.	To examine the factors associated with the development of TBI and TB disease among HIV patients.	- 2,079 PLHIV - PLHIV ≥18 years without active TB or previous history of TB - Exclusion of active TB by clinical/microbiological screening	- TB incidence: • With HAART + TPT: 0.37/100 PY • Without HAART: 1.26/100 PY • TBI+ cases without TPT: higher risk (OR = 6.68; 95%CI: 3.82–11.68) - Risk reduction: TPT associated with lower risk of TB (aHR = 0.34; 95%CI: 0.18–0.65)
Ahmed *et al.*, 2018 [37] Ethiopia	2015 HIV care clinics at government health facilities in northeastern Ethiopia.	To evaluate the incidence of TB and its predictors among PLHIV in government health facilities in northeastern Ethiopia.	- Records of 451 PLHIV - PLHIV without a diagnosis of active TB (WHO symptom- based screening) - No contraindications for isoniazid	- TB incidence: • With IPT: 4 cases/363.6 PY (1.1/100 PY) • Without IPT: 115 cases/1013.8 PY (11.3/100 PY) • Overall protection: 86% lower risk of TB with IPT - Risk reduction: AHR 0.14 (95%CI: 0.05–0.39)
Satiavan *et al.*, 2018 [38] Indonesia	2012- 2016 Medical records from an HIV clinic.	To observe the effect of IPT on the incidence of tuberculosis among PLHIV at the HIV clinic.	- Records of 462 PLHIV - PLHIV ≥15 years - No active TB at the start of TPT - Using ART (with adequate adherence)	- TB incidence: • With TPT: 0.5/100 PY (95%CI: 0.0126–2.027) • Without TPT: 2.4/100 PY (95%CI: 1.515–3.816) - Risk reduction: 79% (IRR = 0.21; 95%CI: 0.023–0.881) - Long- lasting protection: significant effect up to 3 years after TPT
Maharaj *et al.*, 2017 [39] South Africa	2009- 2013 Urban clinical research site in Durban, South Africa.	To describe the outcomes associated with the implementation of IPT in a cohort of TB- infected patients with experience in HIV treatment on ART.	- 212 PLHIV - PLHIV ≥18 years on ART - No signs/symptoms of active TB - No clinical contraindications (history of alcohol abuse, viremia, liver disease/nephropathy) - Exclusion of resistant or previous TB	- TB incidence: • Before TPT: 4.0/100 PY • After TPT: 2.7/100 PY • 9 cases of post- TPT TB (2 during and 7 after completion) - Risk reduction: non- significant 33% reduction in TB incidence (IRR = 0.67; 95% CI: 0.29–1.58; p = 0.362).
Semu *et al.*, 2017 [40] Ethiopia	2007- 2010 14 public health facilities providing ART services in Addis Ababa.	To evaluate the effectiveness of providing IPT for HIV- infected patients in preventing TB occurrence in the Ethiopian context.	- 2,524 PLHIV were followed up for a total of 4,106 PY - PLHIV adults (≥15 years) on HAART or Pre- HAART - No active TB at the start of TPT (negative TB test)	- TB incidence: • With complete TPT: 0.21/100 PY • With incomplete TPT: 0.86/100 PY • Without TPT: 7.18/100 PY - Risk reduction: complete TPT reduced the incidence of TB by 96.3% (aIRR = 0.037; 95%CI: 0.016–0.072) - Duration of protection: Significant protective effect for up to 3 years.
Saito *et al*., 2016 [41] Kenya, Tanzania and Uganda	2003- 2012 35 health facilities in Kenya, Tanzania, and Uganda.	To evaluate the trends in annual TB incidence rates based on healthcare units providing HIV care in public health facilities serving urban and semi- urban populations, and assess patient- and facility- level factors associated with incident TB.	- 168,330 PLHIV - PLHIV in clinical care, without active TB at the beginning of the study	- TB incidence: • With IPT: 2321–4046/100,000 PY (variable by country and ART status) • Without IPT: 3574–5960/100,000 PY (variable by country and ART status) - Risk reduction: 33% reduction in TB risk (adjusted HR: 0.767; 95% CI: 0.728–0.809)
Ayele, Mourik, Bounten, 2015 [42] Ethiopia	2007- 2013 Dilla University Referral Hospital.	To evaluate the effectiveness of IPT in patients receiving ART in a routine care setting.	- 1,922 PLHIV - PLHIV ≥15 years with no history of active TB – Exclusion of active TB by clinical evaluation (no specific criteria until 2010; after 2010, no symptoms such as cough, night sweats, weight loss or fever)	- TB incidence: • With TPT + ART: 0.12/100 PY • Without TPT (ART only): 1.35/100 PY - Risk reduction: 60% lower risk of TB or death with TPT + ART vs. ART alone (aHR = 0.40; 95%CI: 0.18–0.87)
Aquino *et al*., 2015 [43] Brazil	2007- 2012 Two HIV/AIDS referral services.	To dentify the factors associated with the non- initiation of TPT for TBI in PLHIV.	- 232 PLHIV - PLHIV ≥18 years with TST ≥ 5 mm or contact with TB - Exclusion of active TB at baseline	- TB incidence: • With TPT: 0.4/100 PY (95%CI: 0.15–1.44) • Without TPT: 1.2/100 PY (95%CI: 0.39–3.8) • Non- significant difference (p = 0.1325)
Assebe *et al.*, 2015 [44] Ethiopia	2008- 2012 Jimma Hospital.	To compare the TB incidence rate, TB- free survival time, and identify factors associated with TB development among PLHIV in pre- ART care.	- 588 PLHIV - PLHIV ≥15 years in pre- ART follow- up (without ART)	- TB incidence: • With TPT: 2.22/100 PY • Without TPT: 5.06/100 PY - Risk reduction: 50% (aHR = 0.50; 95%CI: 0.26–0.96) - Completion Rate: Not specified (13/294 TB cases in the IPT group vs. 36/294 in the non- IPT group)
Yirdaw *et al.*, 2014 [45] Ethiopia	2007- 2010 The sampling frame consisted of all 20 hospitals in the Nations, Nationalities and Peoples of the South region.	To measure the level of uptake and effectiveness of IPT in reducing the incidence of TB in a cohort of PLHIV enrolled in HIV care.	- 4,484 PLHIV - PLHIV without active TB (excluded after screening) - No contraindications for IPT - No TST	- TB incidence: • With TPT: 0.7/100 PY • Without TPT: 6.1/100 PY - Risk reduction: 65% (aHR = 0.36; 95%CI: 0.19–0.66)
Masini, Sitienei, Weyeinga, 2013 [46] Kenya	2011- 2012 Three HIV care clinics in Eastern Province, Kenya.	To establish rates of treatment completion, loss to follow- up, adverse drug reactions, TB disease and mortality among 606 HIV- infected children during 6 months of IPT.	- 606 PLHIV - HIV+ children (1–14 years) without active TB at the start of TPT. - Exclusion: children <1 year old, suspected TB, active hepatitis, symptoms of peripheral neuropathy, history of TB in the last 2 years or poor adherence to ART.	- TB incidence: • With TPT: 3% (18/606) developed active TB (median diagnosis at 3 weeks after starting TPT). • Without TPT: Data not available. − 72% of TB cases were diagnosed in the first 5 weeks, suggesting previous infection not detected in the initial screening.
Sibanda *et al*., 2013 [47] Botswana	2004- 2006 Eight public health clinics in Gaboronea and Francistown, Botswana.	To describe the characteristics and outcomes of TB cases in HIV- infected adults exposed to IPT, with access to ART and anti- TB treatment.	- 1,995 PLHIV - PLHIV ≥18 years – No signs or symptoms of active TB (cough, weight loss, fever, etc.) - No history of TB treatment in the last 3 years - No radiographic changes suspicious of TB (unless there was a previous history of TB or pneumonia)	- TB incidence: • With TPT: 3% (75/1995) developed active TB. • Without TPT: Data not available. - Of the total number of people who received TPT, 619 had TB respiratory symptoms. The average time to start anti- tuberculosis treatment was 12 months after the end of IPT.
Martínez- Pino *et al*., 2013 [48] Spain	2004- 2009 20 Hospitals	To describe the incidence of TB and factors related to the development of TB after treatment for TBI in HIV- 1 infected patients in the era of highly active ART.	- 7,902 PLHIV - HIV+ patients with positive TST (induration ≥5 mm). - No active TB at the start of TPT. - Exclusion: history of previous TB or current TB diagnosis.	- TB incidence: • With TPT: 1.75 cases/100 PY. • Without TPT: 6 cases/100 PY. - Risk reduction: 70.8% (RR = 0.29) in TST + . - Risk factors: Age < 35 years (HR 6.14; 95%CI 1.12–33.73); CD4+ < 200 cells/μl (HR 5.64; 95%CI 1.34–23.70).
Frigati *et al.*, 2011 [49] South Africa	2003- 2007 Two centers in Cape Town, South Africa	To investigate the combined effect of preventive therapy with isoniazid and ART on the risk of TB in HIV- infected children.	- 298 children - HIV+ children (>8 weeks old) - Weight >2.5 kg - No active TB at onset (clinical, radiological and microbiological assessment) - Exclusion: severe anemia, neutropenia, renal failure	- TB incidence: • With TPT: 13% (39/298) developed active TB. • Without TPT: Data not available. - Risk reduction: TPT alone: HR = 0.22 (95%CI: 0.09–0.53); TPT + ART: HR = 0.11 (95%CI: 0.04–0.32) vs. placebo
Golub *et al.*, 2009 [50] South Africa	2003- 2007 HIV clinics.	To analyze data from two clinical cohorts of HIV- infected adults in South Africa.	- 2,778 PLHIV were followed up for a total of 4,287 PY - PLHIV without active TB (excluded if previously diagnosed or within 60 days of entry) - At one of the sites, TST > 5 mm was required	- TB incidence: • With TPT + ART: 1.1/100 PY • Without TPT (ART only): 4.6/100 PY - Risk reduction: 89% (aHR = 0.11; 95%CI: 0.02–0.78)
Gourevitch et al., 1999 United States of America [51]	1985- 1996 HIV- positive drug users enrolled in a methadone maintenance treatment program with on- site primary care, located in the Bronx, New York.	To define the efficacy of chemoprophylaxis, outside of a clinical trial setting, in preventing TB among TB- reactive and anergic HIV- infected drug users at high risk of developing active TB.	- 155 PLHIV - TST reaction (PPD ≥ 5 mm) - No history of active TB or previous TPT	- TB incidence: • With complete TPT: 0.51/100 person- years • Without TPT: 2.07/100 person- years

Caption: TB = tuberculosis; TPT = tuberculosis preventive treatment; IPT = isoniazid preventive therapy; HIV = human immunodeficiency virus; AIDS = acquired immunodeficiency syndrome; TBI = tuberculosis infection; PY = person- years; HR = hazard ratio; aHR = adjusted hazard ratio; ART = antiretroviral therapy; PLHIV = people living with HIV; TST = tuberculin skin test; TARI = tuberculosis and HIV/AIDS referral and information clinics; HAART = highly active antiretroviral therapy.

Source: the authors.

The 34 articles were published between 1999 and 2024, with 82% conducted in Sub-Saharan Africa: Ethiopia contributed with 13 studies [18,23,27,29,31–34,37,40,42,44,45], South Africa with five [24,26,39,49,50], Kenya with three [21,41,46], Tanzania with three [25,35,41], Uganda with two [22,41], Botswana with one [47] and Zambia with one [20]. In addition, Brazil contributed with three studies [19,28,43] and Spain with one [48], the United States with one [51], Hong Kong with one [36], India with one [30], and Indonesia with one [38] contributed with 1 study each. S1 Fig (Supplementary Information) shows the geographical distribution of the studies. Only one study [18] reported a cohort using rifapentine + isoniazid, in addition to isoniazid alone, all other cohorts used isoniazid TPT. Their settings feature a variety of ART or HIV centers, ranging from clinics to hospitals, reference centers, and specialized health units. All reported a risk reduction of TB, although with varying degrees of effect.

The random-effects meta-analysis included 13 studies evaluating the protective effect of the intervention in tuberculosis incidence, 11 conducted in adults and two in children. The forest plot visually displays a pooled HR of 0.36 (95% CI: 0.27–0.48), indicating a consistent protective effect across most studies, despite substantial heterogeneity (I² = 87.7%, τ² = 0.1613, p < 0.001).

**Fig 2:**
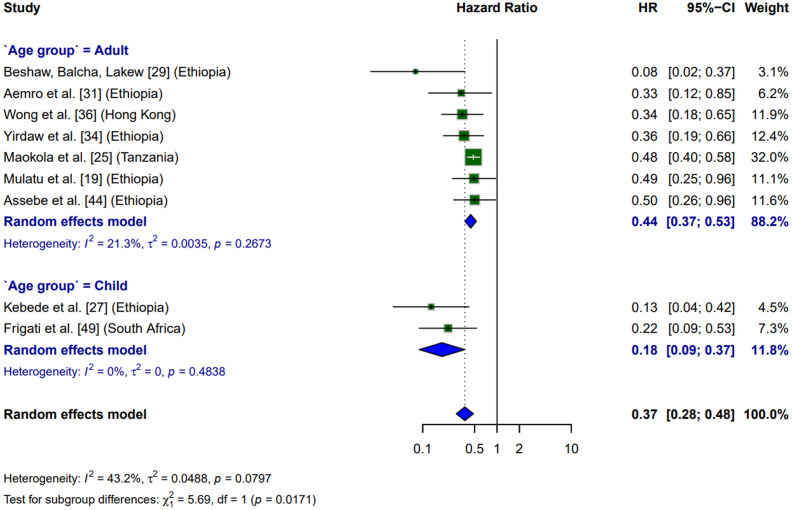
Forest plot of hazard ratios for incidence to active tuberculosis among people living with HIV undergoing preventive therapy, stratified by age group.

Influence analysis identified four outlier studies: Saito et al. [41] (Cook’s d = 0.13), Golub et al. [50] (Cook’s d = 0.03), Atey et al. [32] (Cook’s d = 0.009), and Russom et al. [24] (Cook’s d = 0.29). After excluding these studies, heterogeneity was substantially reduced (I² = 43%, τ² = 0.0488), with a resulting 63% reduction in the risk of tuberculosis incidence following preventive treatment (HR = 0.37, 95% CI: 0.28–0.48). Although the studies by Maokola et al. [25] and Frigati et al. [49] demonstrated high influence, their exclusion did not meaningfully change the overall pooled HR (Fig 2).

### Subgroup analysis

There were stronger protective effects in children compared to adults (p = 0.01, Fig 2). Among adults, seven studies indicated a median risk reduction of 56% (pooled HR: 0.44; 95% CI: 0.37–0.53), with low between-study heterogeneity (I² = 21.3%). The study by Maokola et al. [25] in Tanzania contributed the largest weight to the analysis (32.0%) and reported a consistent 52% risk reduction (HR: 0.48; 95% CI: 0.40–0.58). In contrast, Beshaw et al. [29] in Ethiopia demonstrated the most pronounced protective effect (92% reduction, HR: 0.08; 95% CI: 0.02–0.37), although with lower precision, accounting for 3.1% of the weight. Such variability may reflect regional differences in intervention implementation or underlying population characteristics (Fig 2).

In children, the two available studies demonstrated a markedly higher protective effect, with a pooled hazard ratio of 0.18 (95% CI: 0.09–0.37), corresponding to an 82% reduction in the risk of tuberculosis. Kebede et al. [27] in Ethiopia reported an 87% reduction (HR: 0.13; 95% CI: 0.04–0.42), while Frigati et al. [49] in South Africa observed a 78% reduction (HR: 0.22; 95% CI: 0.09–0.53). The complete homogeneity between pediatric studies (I² = 0%) reinforces the robustness of this protective effect (Fig 2).

No significant difference in the protective effect of TPT was found among studies according to ART use (p > 0.05) in the meta-regression analysis.

### Quality of the studies included in the review

The methodological quality scores using the NOS [17] ranged from three to eight points, with a maximum possible score of nine. The majority of studies (n = 33) were classified as having a moderate or low risk of methodological or reporting bias (S4 Table).

Specifically, 18 studies [18,19,22,24,25,30–33,36,37,39–41,43–46] scored seven or eight points and were therefore classified as having a low risk of bias. Sixteen studies [20,21,23,26,27,29,34,35,38,42,47–51] scored four, five, or six points and were considered to have a moderate risk of bias, while only one study was classified as having a high risk of bias [28], with a score of 3/9 (S4 Table).

**Fig 3:**
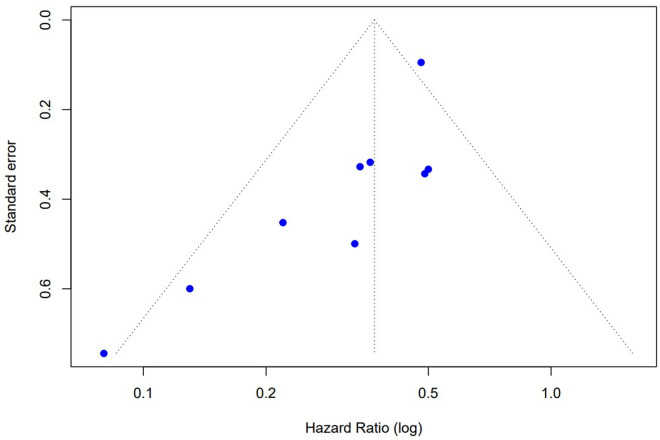
Funnel plot for assessing potential publication bias among studies included in the meta-analysis on tuberculosis incidence risk among people living with HIV/AIDS after tuberculosis preventive treatment.

Fig 3 shows the funnel plot of the 9 studies included in the meta-analysis. Visually, no evident asymmetry was observed among the points, suggesting a low likelihood of publication bias.

Each blue dot represents a study included in the meta-analysis. The vertical dashed line indicates the pooled hazard ratio (log-transformed), and the diagonal lines represent the 95% confidence limits expected in the absence of bias.

## Discussion

This systematic review and metanalysis of observational cohort studies reinforces the effectiveness of TPT in real-world settings, showing a 60% overall risk reduction, ranging from 50 to 92%. Our study corroborates the findings of previous meta-analyses that have evaluated the efficacy of TPT in reducing the incidence of active tuberculosis among PLHIV, using data solely from mathematical models [10] and clinical trials [11]. Additionally, our global results align with the protective effect observed in Ethiopian programmatic settings [12], who reported a 74% TB risk reduction among PLHIV receiving ART with IPT. This consistency across diverse contexts strengthens the case for TPT’s broad applicability beyond controlled research environments. To our knowledge, our study is the first to explore age-specific differences on a global scale.

The consistency of the protective effect of TPT, even in the presence of initial heterogeneity across studies reinforces the robustness of this review’s findings. Through sensitivity analysis, four studies with high statistical impact were identified and excluded, without compromising the direction or magnitude of the estimated association. This stability suggests that despite contextual and methodological variations across studies, the benefit of TPT remains clear and reproducible, reinforcing its relevance for practice in diverse settings.

The risk of rapid incidence to active tuberculosis is higher in children than in adults, mainly in children under five [52], raising special concern in this age group. The greater magnitude of TPT protective effect found in children living with HIV in the present review may be due to the higher initial risk but also to earlier intervention, lower burden of comorbidities, or better treatment adherence when supervised, as suggested in household contacts [53].

Strong protection in high-burden countries such as Ethiopia and South Africa support the hypothesis of more intense protection in higher-risk populations. However, there was great heterogeneity in the effect even among the high-burden countries. According to previous studies, this variability may be attributed to differences in study design, populations assessed, TPT implementation strategies, ART adherence, and individual immunological profiles, including hemoglobin levels, CD4 + T cell counts, and viral load, rather than differences between countries alone [19,54]. It was beyond the scope of the current study to analyze the reasons for heterogeneity.

Operational challenges, such as difficulties in ruling out active tuberculosis before starting TPT, fear of adverse reactions, limited adherence to guidelines and lack of drugs, have been noted as possible barriers to the effective implementation of TPT [20,21,46,55]. These limitations may partially explain the heterogeneity observed and suggest that the protective effect of TPT could be even greater in the absence of such restrictions.

In our meta-analysis, no significant association was found between tuberculosis incidence and ART use among individuals who underwent TPT. This suggests that the protective effect of TPT persists regardless of ART status. Nonetheless, this finding should be interpreted with caution, as only a few studies included participants not on ART or clearly reported ART status as an inclusion criterion [44,45].

Our review also points out the lack of knowledge on TPT protection in low-burden settings, and to the paucity of comparison of the effectiveness of different TPT regimens. Apart from one study [18], all others were cohorts of isoniazid TPT.

One of the limitations of this review is the residual heterogeneity among studies involving adults (I² = 21.3%), which may reflect differences in eligibility criteria, TPT regimens, or the quality of active tuberculosis screening. Furthermore, the smaller number of pediatric studies limits more detailed subgroup analyses and points to the need for additional research focused on children. Factors such as immune status and interaction with ART should be investigated in future studies to optimize prevention strategies.

This study not only contributes to the understanding of the dynamics associated with tuberculosis-HIV co-infection, but also provides subsidies to improve global strategies for the prevention and control of tuberculosis among PLHIV, with an emphasis on an integrated and evidence-based approach. Additionally, the consistency of the findings, even after the exclusion of influential studies in the sensitivity analysis, reinforces the robustness and reliability of the estimated effect.

## Conclusion

This systematic review and meta-analysis of cohort studies provides evidence of the effectiveness of TPT in reducing the incidence of active tuberculosis among PLHIV, with conmore pronounced benefits observed in children. Although some variability was observed among adult studies, the overall findings confirm the protective benefit previously demonstrated in clinical trials and show the feasibility of this strategy. These results reinforce global recommendations for broad implementation and scale up of TPT among PLHIV and highlight the importance of age-specific strategies.

Finally, the expanded implementation of TPT, particularly among pediatric populations, may contribute significantly to global tuberculosis elimination goals.

## Supporting information

S1 Table PRISMA Checklist. (PDF)

S2 Table Search terms. (PDF)

S3 Table Excluded articles. (PDF)

S4 Table Methodological Quality Assessment. (PDF)

S1 Fig Geographical distribution of included studies. (PDF)
