# Supplementary material for: Effectiveness of tuberculosis preventive treatment on disease incidence among people living with HIV/AIDS: A systematic review and meta-analysis
Source: PLoS One. 2025 Aug 26;20(8):e0330208. doi: 10.1371/journal.pone.0330208 (PMC12380327; doi:10.1371/journal.pone.0330208)
Supplement: S2 Table — (PDF) [file pone.0330208.s002.pdf]

Table S2. Search terms

PubMed

Mnemonic		Descriptors	Records retrieved
Population	#1	"hiv"[MeSH Terms] OR "hiv"[All Fields] OR "HIV Infection"[All Fields] OR "HIV Infections"[All Fields] OR "Human Immunodeficiency Virus"[All Fields] OR "Human Immunedeficiency Virus"[All Fields] OR "human immuno deficiency virus"[All Fields] OR "human immune deficiency virus"[All Fields] OR "human immuno deficiency virus"[All Fields] OR "human immune deficiency virus"[All Fields] OR ("Acquired Immunodeficiency Syndrome"[MeSH Terms] OR ("acquired"[All Fields] AND "immunodeficiency"[All Fields] AND "syndrome"[All Fields]) OR "Acquired Immunodeficiency Syndrome"[All Fields] OR "aids"[All Fields]) OR "Acquired Immunodeficiency Syndrome"[All Fields] OR "Acquired Immunedeficiency Syndrome"[All Fields] OR "acquired immuno deficiency syndrome"[All Fields] OR "acquired immune deficiency syndrome"[All Fields] OR "acquired immune deficiency syndrome"[All Fields] OR "acquired immuno deficiency syndrome"[All Fields]	**569,542**
Exposure	#2	"Tuberculosis Preventive Treatment"[All Fields] OR "tb Preventive Treatment"[All Fields] OR (("therapeutics"[MeSH Terms] OR "therapeutics"[All Fields] OR "treatments"[All Fields] OR "therapy"[MeSH Subheading] OR "therapy"[All Fields] OR "treatment"[All Fields] OR "treatment s"[All Fields]) AND ("tuberculosis"[MeSH Terms] OR "tuberculosis"[All Fields] OR ("tuberculosis"[All Fields] AND "infection"[All Fields]) OR "tuberculosis infection"[All Fields] OR "latent tuberculosis"[MeSH Terms] OR ("latent"[All Fields] AND "tuberculosis"[All Fields]) OR "latent tuberculosis"[All Fields])) OR "Treatment of tb Infection"[All Fields] OR (("latent"[All Fields] OR "latently"[All Fields] OR "latents"[All Fields]) AND ("mycobacterium tuberculosis"[MeSH Terms] OR ("mycobacterium"[All Fields] AND "tuberculosis"[All Fields]) OR "mycobacterium tuberculosis"[All Fields] OR "m tuberculosis"[All Fields]) AND ("infect"[All Fields] OR "infectability"[All Fields] OR "infectable"[All Fields] OR "infectant"[All Fields] OR "infectants"[All Fields] OR "infected"[All Fields] OR "infecteds"[All Fields] OR "infectibility"[All Fields] OR "infectible"[All Fields] OR "infecting"[All Fields] OR "infection s"[All Fields] OR "infections"[MeSH Terms] OR "infections"[All Fields] OR "infection"[All Fields] OR "infective"[All Fields] OR "infectiveness"[All Fields] OR "infectives"[All Fields] OR "infectivities"[All Fields] OR "infects"[All Fields] OR "pathogenicity"[MeSH Subheading] OR "pathogenicity"[All Fields] OR "infectivity"[All Fields]) AND ("therapeutics"[MeSH Terms] OR "therapeutics"[All Fields] OR "treatments"[All Fields] OR "therapy"[MeSH Subheading] OR "therapy"[All Fields] OR "treatment"[All Fields] OR "treatment s"[All Fields])) OR (("latent"[All Fields] OR "latently"[All Fields] OR "latents"[All Fields]) AND ("tuberculosis"[MeSH Terms] OR "tuberculosis"[All Fields] OR ("mycobacterium"[All Fields] AND "tuberculosis"[All Fields] AND "infection"[All Fields]) OR "mycobacterium tuberculosis infection"[All Fields]) AND ("therapeutics"[MeSH Terms] OR "therapeutics"[All Fields] OR "treatments"[All Fields] OR "therapy"[MeSH Subheading] OR "therapy"[All Fields] OR "treatment"[All Fields] OR "treatment s"[All Fields])) OR (("therapeutics"[MeSH Terms] OR "therapeutics"[All Fields] OR "treatments"[All Fields] OR "therapy"[MeSH Subheading] OR "therapy"[All Fields] OR "treatment"[All Fields] OR "treatment s"[All Fields]) AND ("latent tuberculosis"[MeSH Terms] OR ("latent"[All Fields] AND "tuberculosis"[All Fields]) OR "latent tuberculosis"[All Fields] OR ("latent"[All Fields] AND "tuberculosis"[All Fields] AND "infection"[All Fields]) OR "latent tuberculosis infection"[All Fields])) OR (("therapeutics"[MeSH Terms] OR "therapeutics"[All Fields] OR "treatments"[All Fields] OR "therapy"[MeSH Subheading] OR "therapy"[All Fields] OR "treatment"[All Fields] OR "treatment s"[All Fields]) AND ("latent"[All Fields] OR "latently"[All Fields] OR "latents"[All Fields]) AND "tb"[All Fields] AND ("infect"[All Fields] OR "infectability"[All Fields] OR "infectable"[All Fields] OR "infectant"[All Fields] OR "infectants"[All Fields] OR "infected"[All Fields] OR "infecteds"[All Fields] OR "infectibility"[All Fields] OR "infectible"[All Fields] OR "infecting"[All Fields] OR "infection s"[All Fields] OR "infections"[MeSH Terms] OR "infections"[All Fields] OR "infection"[All Fields] OR "infective"[All Fields] OR "infectiveness"[All Fields] OR "infectives"[All Fields] OR "infectivities"[All Fields] OR "infects"[All Fields] OR "pathogenicity"[MeSH Subheading] OR "pathogenicity"[All Fields] OR "infectivity"[All Fields])) OR "tuberculosis preventive therapy"[All Fields] OR "tb preventive therapy"[All Fields] OR "Latent Tuberculosis treatment"[All Fields] OR "Latent tb treatment"[All Fields] OR "Latent Tuberculosis therapy"[All Fields] OR (("latent"[All Fields] OR "latently"[All Fields] OR "latents"[All Fields]) AND "tb"[All Fields] AND ("therapeutics"[MeSH Terms] OR "therapeutics"[All Fields] OR "therapies"[All Fields] OR "therapy"[MeSH Subheading] OR "therapy"[All Fields] OR "therapy s"[All Fields] OR "therapys"[All Fields])) OR "tuberculosis infection treatment"[All Fields] OR "tb infection treatment"[All Fields] OR "tuberculosis infection therapy"[All Fields] OR "tb infection therapy"[All Fields] OR "isoniazid preventive therapy"[All Fields] OR "isoniazid preventive treatment"[All Fields] OR "ipt"[All Fields]	**154,195**

Outcome	#3	"tb disease progression"[All Fields] OR "tuberculosis disease progression"[All Fields] OR "tb disease development"[All Fields] OR "tuberculosis disease development"[All Fields] OR (("develop"[All Fields] OR "develope"[All Fields] OR "developed"[All Fields] OR "developer"[All Fields] OR "developer s"[All Fields] OR "developers"[All Fields] OR "developing"[All Fields] OR "developments"[All Fields] OR "develops"[All Fields] OR "growth and development"[MeSH Subheading] OR ("growth"[All Fields] AND "development"[All Fields]) OR "growth and development"[All Fields] OR "development"[All Fields]) AND "tb"[All Fields]) OR (("develop"[All Fields] OR "develope"[All Fields] OR "developed"[All Fields] OR "developer"[All Fields] OR "developer s"[All Fields] OR "developers"[All Fields] OR "developing"[All Fields] OR "developments"[All Fields] OR "develops"[All Fields] OR "growth and development"[MeSH Subheading] OR ("growth"[All Fields] AND "development"[All Fields]) OR "growth and development"[All Fields] OR "development"[All Fields]) AND ("tuberculosi"[All Fields] OR "tuberculosis"[MeSH Terms] OR "tuberculosis"[All Fields] OR "tuberculoses"[All Fields] OR "tuberculosis s"[All Fields])) OR "developing tb"[All Fields] OR "developing tuberculosis"[All Fields] OR "developed tb"[All Fields] OR "developed tuberculosis"[All Fields] OR "tb incidence"[All Fields] OR "tb incident cases"[All Fields] OR "incident tb"[All Fields] OR "incident tuberculosis"[All Fields] OR "tb disease development"[All Fields]	**62,314**
	**#4**	**#1 AND #2 AND #3**	**7,478**

Source: the authors.

Embase

**Mnemonic**		**Descriptors**	**Records retrieved**
Population	#1	('hiv'/exp OR hiv OR 'hiv infection'/exp OR 'hiv infection' OR 'hiv infections'/exp OR 'hiv infections' OR 'human immunodeficiency virus'/exp OR 'human immunodeficiency virus' OR 'human immunedeficiency virus' OR 'human immuno-deficiency virus'/exp OR 'human immuno-deficiency virus' OR 'human immune-deficiency virus' OR 'human immuno deficiency virus'/exp OR 'human immuno deficiency virus' OR 'human immune deficiency virus' OR 'aids'/exp OR aids OR 'acquired immunodeficiency syndrome'/exp OR 'acquired immunodeficiency syndrome' OR 'acquired immunedeficiency syndrome' OR 'acquired immuno-deficiency syndrome'/exp OR 'acquired immuno-deficiency syndrome' OR 'acquired immune-deficiency syndrome'/exp OR 'acquired immune-deficiency syndrome' OR 'acquired immune deficiency syndrome'/exp OR 'acquired immune deficiency syndrome' OR 'acquired immuno deficiency syndrome'/exp OR 'acquired immuno deficiency syndrome') AND ([embase]/lim OR [preprint]/lim)	**852,691**
Exposure	#2	('tuberculosis preventive treatment' OR 'tb preventive treatment' OR 'treatment of tuberculosis infection' OR 'treatment of tb infection' OR 'latent m. tuberculosis infection treatment' OR 'latent mycobacterium tuberculosis infection treatment' OR 'treatment of latent tuberculosis infection' OR 'treatment of latent tb infection' OR 'tuberculosis preventive therapy' OR 'tb preventive therapy' OR 'latent tuberculosis treatment' OR 'latent tb treatment' OR 'latent tuberculosis therapy' OR 'latent tb therapy' OR 'tuberculosis infection treatment' OR 'tb infection treatment' OR 'tuberculosis infection therapy' OR 'tb infection therapy' OR 'isoniazid preventive therapy'/exp OR 'isoniazid preventive therapy' OR 'isoniazid preventive treatment' OR ipt) AND ([embase]/lim OR [preprint]/lim)	**5,490**
Outcome	#3	('tb disease progression' OR 'tuberculosis disease progression' OR 'tuberculosis disease development' OR 'develop tb' OR 'develop tuberculosis' OR 'developing tb' OR 'developing tuberculosis' OR 'developed tb' OR 'developed tuberculosis' OR 'tb incidence' OR 'tb incident cases' OR 'incident tb' OR 'incident tuberculosis' OR 'tb disease development') AND ([embase]/lim OR [preprint]/lim)	**4,534**
	**#4**	**#1 AND #2 AND #3**	**401**

Source: the authors.

Scopus

Mnemonic		Descriptors	Records retrieved
Population	#1	TITLE-ABS-KEY (hiv OR "HIV Infection" OR "HIV Infections" OR "Human Immunodeficiency Virus" OR "Human Immunedeficiency Virus" OR "Human Immuno-deficiency Virus" OR "Human Immune-deficiency Virus" OR "Human Immuno deficiency Virus" OR "Human Immune deficiency Virus" OR aids OR "Acquired Immunodeficiency Syndrome" OR "Acquired Immunedeficiency Syndrome" OR "Acquired immuno-deficiency syndrome" OR "Acquired immune-deficiency syndrome" OR "Acquired Immune Deficiency Syndrome" OR "Acquired Immuno Deficiency Syndrome")	**779,202**
Exposure	#2	TITLE-ABS-KEY ("Tuberculosis Preventive Treatment" OR "tb Preventive Treatment" OR "Treatment of Tuberculosis Infection" OR "Treatment of tb Infection" OR "Latent M. tuberculosis Infection Treatment" OR "Latent Mycobacterium tuberculosis Infection Treatment" OR "Treatment of latent tuberculosis infection" OR "Treatment of latent tb infection" OR "tuberculosis preventive therapy" OR "tb preventive therapy" OR "Latent Tuberculosis treatment" OR "Latent tb treatment" OR "Latent Tuberculosis therapy" OR "Latent tb therapy" OR "tuberculosis infection treatment" OR "tb infection treatment" OR "tuberculosis infection therapy" OR "tb infection therapy" OR "isoniazid preventive therapy" OR "isoniazid preventive treatment" OR ipt)	**10,053**
Interest	#3	TITLE-ABS-KEY ("tb disease progression" OR "tuberculosis disease progression" OR "tb disease development" OR "tuberculosis disease development" OR "develop tb" OR "develop tuberculosis" OR "developing tb" OR "developing tuberculosis" OR "developed tb" OR "developed tuberculosis" OR "tb incidence" OR "tb incident cases" OR "incident tb" OR "incident tuberculosis" OR "tb disease development")	**4,366**
	**#4**	**#1 AND #2 AND #3**	**220**

Source: the authors.

Web of Science

Mnemonic		Descriptors	Records retrieved
Population	#1	HIV OR "HIV Infection" OR "HIV Infections" OR "Human Immunodeficiency Virus" OR "Human Immunedeficiency Virus" OR "Human Immuno-deficiency Virus" OR "Human Immune-deficiency Virus" OR "Human Immuno deficiency Virus" OR "Human Immune deficiency Virus" OR AIDS OR "Acquired Immunodeficiency Syndrome" OR "Acquired Immunedeficiency Syndrome" OR "Acquired immuno-deficiency syndrome" OR "Acquired immune-deficiency syndrome" OR "Acquired Immune Deficiency Syndrome" OR "Acquired Immuno Deficiency Syndrome"	**1,217,987**
Exposure	#2	"Tuberculosis Preventive Treatment" OR "tb Preventive Treatment" OR "Treatment of Tuberculosis Infection" OR "Treatment of tb Infection" OR "Latent M. tuberculosis Infection Treatment" OR "Latent Mycobacterium tuberculosis Infection Treatment" OR "Treatment of latent tuberculosis infection" OR "Treatment of latent tb infection" OR "tuberculosis preventive therapy" OR "tb preventive therapy" OR "Latent Tuberculosis treatment" OR "Latent tb treatment" OR "Latent Tuberculosis therapy" OR "Latent tb therapy" OR "tuberculosis infection treatment" OR "tb infection treatment" OR "tuberculosis infection therapy" OR "tb infection therapy" OR "isoniazid preventive therapy" OR "isoniazid preventive treatment" OR ipt	**12,749**

Interest	#3	"tb disease progression" OR "tuberculosis disease progression" OR "tb disease development" OR "tuberculosis disease development" OR "develop tb" OR "develop tuberculosis" OR "developing tb" OR "developing tuberculosis" OR "developed tb" OR "developed tuberculosis" OR "tb incidence" OR "tb incident cases" OR "incident tb" OR "incident tuberculosis" OR "tb disease development"	**3,416**
	**#4**	**#1 AND #2 AND #3**	**227**

Source: the authors.

LILACS

**Mnemonic**		**Descriptors**	**Records retrieved**
Population	#1	HIV OR "HIV Infection" OR "HIV Infections" OR "Human Immunodeficiency Virus" OR "Human Immunedeficiency Virus" OR "Human Immuno-deficiency Virus" OR "Human Immune-deficiency Virus" OR "Human Immuno deficiency Virus" OR "Human Immune deficiency Virus" OR AIDS OR "Acquired Immunodeficiency Syndrome" OR "Acquired Immunedeficiency Syndrome" OR "Acquired immuno-deficiency syndrome" OR "Acquired immune-deficiency syndrome" OR "Acquired Immune Deficiency Syndrome" OR "Acquired Immuno Deficiency Syndrome" OR "Infecção pelo HIV" OR "Infecções pelo HIV" OR "Vírus da Imunodeficiência Humana" OR "Vírus da Imunodeficiência Adquirida" OR "Síndrome da Imunodeficiência Adquirida" OR VIH OR "Infección por VIH" OR "Infecciones por VIH" OR "Virus de inmunodeficiencia humana" OR "Virus de inmunodeficiencia adquirida" OR SIDA OR "Síndrome de inmunodeficiencia adquirida"	**27,777**
Exposure	#2	"Tuberculosis Preventive Treatment" OR "tb Preventive Treatment" OR "Treatment of Tuberculosis Infection" OR "Treatment of tb Infection" OR "Latent M. tuberculosis Infection Treatment" OR "Latent Mycobacterium tuberculosis Infection Treatment" OR "Treatment of latent tuberculosis infection" OR "Treatment of latent tb infection" OR "tuberculosis preventive therapy" OR "tb preventive therapy" OR "Latent Tuberculosis treatment" OR "Latent tb treatment" OR "Latent Tuberculosis therapy" OR "Latent tb therapy" OR "tuberculosis infection treatment" OR "tb infection treatment" OR "tuberculosis infection therapy" OR "tb infection therapy" OR "isoniazid preventive therapy" OR "isoniazid preventive treatment" OR ipt OR "Tratamento preventivo de tuberculose" OR "Tratamento preventivo de tb" OR "Tratamento de infecção por tuberculose" OR "Tratamento de infecção por tb" OR "Tratamento de infecção latente por M. tuberculosis" OR "Tratamento de infecção latente por Mycobacterium tuberculosis" OR "Tratamento de infecção latente por tuberculose" OR "Tratamento de infecção latente de tuberculose" OR "terapia preventiva de tuberculose" OR "terapia preventiva de tb" OR "Tratamento de tuberculose latente" OR "Tratamento de tb latente" OR "Terapia de tuberculose latente" OR "Terapia de tb latente" OR "tratamento de infecção de tuberculose" OR "tratamento para infecção por tuberculose" OR "terapia para tuberculose infecção" OR "terapia para infecção por tb" OR "terapia preventiva com isoniazida" OR "tratamento preventivo com isoniazida" OR "Tratamiento preventivo de la tuberculosis" OR "Tratamiento de la infección por tuberculosis" OR "Tratamiento de la infección latente por M. tuberculosis" OR "Tratamiento de la infección por Mycobacterium tuberculosis latente" OR "Tratamiento de la infección por tuberculosis latente" OR "terapia preventiva de la tuberculosis" OR "Tratamiento de la tuberculosis latente" OR "Terapia de la tuberculosis latente" OR "tratamiento de la infección de tuberculosis" OR "tratamiento de la infección por tuberculosis" OR "terapia de la infección por tuberculosis" OR "terapia de la infección de tuberculosis" OR "terapia preventiva con isoniazida" OR "tratamiento preventivo con isoniazida"	**113**

Interest	#3	"tb disease progression" OR "tuberculosis disease progression" OR "tb disease development" OR "tuberculosis disease development" OR "develop tb" OR "develop tuberculosis" OR "developing tb" OR "developing tuberculosis" OR "developed tb" OR "developed tuberculosis" OR "tb incidence" OR "tb incident cases" OR "incident tb" OR "incident tuberculosis" OR "tb disease development" OR "progressão da doença tuberculosa" OR "desenvolvimento da doença tuberculosa" OR "desenvolver tuberculose" OR "desenvolver tb" OR "desenvolvimento de tuberculose" OR "desenvolvimento de tb" OR "desenvolveu tuberculose" OR "incidência de tuberculose" OR "casos incidentes de tuberculose" OR "incidente de tuberculose" OR "tuberculose incidente" OR "desenvolvimento de tuberculose doença" OR "desenvolvimento de TB doença" OR "progresión de la tuberculosis" OR "desarrollo de la tuberculosis" OR "desarrollar tuberculosis" OR "incidencia de tuberculosis" OR "casos incidentes de tuberculosis" OR "tuberculosis incidente" OR "desarrollo de la tuberculosis enfermedad"	**347**
	**#4**	**#1 AND #2 AND #3**	**4**

Source: the authors.
