# Supplementary material for: Effectiveness of tuberculosis preventive treatment on disease incidence among people living with HIV/AIDS: A systematic review and meta-analysis
Source: PLoS One. 2025 Aug 26;20(8):e0330208. doi: 10.1371/journal.pone.0330208 (PMC12380327; doi:10.1371/journal.pone.0330208)
Supplement: S3 Table — (PDF) [file pone.0330208.s003.pdf]

Table S3. Excluded articles

Title	Author, year	Excluded studies	Specific reason for exclusion	Responsible for data extraction
Factors associated with tuberculosis among PLHIV: An observational study in a Government Medical College in West Bengal, India	Gosh et al., 2024	Another type of study	Cross-sectional	GCL
Long-Term Protective Effect of Tuberculosis Preventive Therapy in a Medium/High Tuberculosis Incidence Setting	Teixeira et al., 2024	Another type of study	Randomized clinical trial	JNBSJ
Prevalence and associated factors of tuberculosis among isoniazid users and non-users of HIV patients in Dessie, Ethiopia	Mebratu et al., 2022	Another type of study	Cross-sectional	QRF
Isoniazid preventive therapy and tuberculosis transcriptional signatures in people with HIV	Valinetz et al., 2022	Another type of study	Cross-sectional	LKAA
Active TB infection and its associated factors among HIV-1 infected patients at Jimma medical center, Southwest Ethiopia	Mulugeta et al., 2021	Another type of study	Cross-sectional	QRF
Pulmonary tuberculosis screening in anti-retroviral treated adults living with HIV in Kenya	Gersh et al., 2021	Another type of study	Cross-sectional	JNBSJ
Utilization of isoniazid prophylaxis therapy and its associated factors among HIV positive clients taking antiretroviral therapy at Fre Semaetat primary hospital, Hawzien districts, Tigrai, Northern Ethiopia	Legese et al., 2020	Another type of study	Cross-sectional	QRF
Infecção latente por tuberculose entre pessoas com HIV/AIDS, fatores associados e progressão para doença ativa em município no Sul do Brasil	Santos et al., 2017	Another type of study	Cross-sectional	GCL
Urine Lipoarabinomannan Testing in Adults With Advanced Human Immunodeficiency Virus in a Trial of Empiric Tuberculosis Therapy	Matoga et al., 2021	Another type of study	Randomized clinical trial	GCL
Inference without randomization or ignorability: A stability-controlled quasi-experiment on the prevention of tuberculosis	Hazlett et al., 2020	Another type of study	Causal inference study with potential results approach	QRF
Implementing an isoniazid preventive therapy program for people living with HIV in Thailand	Danyuttapolchai et al., 2017	Another type of study	Implementation study	QRF
Impact of isoniazid preventive therapy for HIV-infected adults in Rio de Janeiro, Brazil: an epidemiological model	Dowdy et al., 2014	Another type of study	Mathematical model based on a randomized clinical trial	LKAA
Effect of improved tuberculosis screening and isoniazid preventive therapy on incidence of tuberculosis and death in patients with HIV in clinics in Rio de Janeiro, Brazil: a stepped wedge, cluster-randomised trial	Durovni et al., 2013	Another type of study	Randomized clinical trial	QRF

Isoniazid Adherence Reduces Mortality and Incident Tuberculosis at 96 Weeks Among Adults Initiating Antiretroviral Therapy With Advanced Human Immunodeficiency Virus in Multiple High-Burden Settings	Gupta et al., 2022	Another type of study	Randomized clinical trial	QRF
6-month versus 36-month isoniazid preventive treatment for tuberculosis in adults with HIV infection in Botswana: a randomised, double-blind, placebo-controlled trial	Samandari et al, 2011	Another type of study	Randomized clinical trial	JNBSJ
The implementation of isoniazid preventive therapy in HIV clinics: the experience from the TB/HIV in Rio (THRio) study	.Durovni et al., 2010	Another type of study	Randomized clinical trial	JNBSJ
Primary and secondary tuberculosis preventive treatment in HIV clinics: Simulating alternative strategies	Basu et al., 2009	Another type of study	Mathematical simulation study	GCL
Randomised controlled trial of isoniazid preventive therapy in South African adults with advanced HIV disease	Mohammed et al., 2007	Another type of study	Randomized clinical trial	JNBSJ
Risk Factors for Development of Tuberculosis after Isoniazid Chemoprophylaxis in Human Immunodeficiency Virus– Infected Patients	Casado et al, 2002	Another type of study	Report	GCL
Isoniazid preventive therapy for tuberculosis in HIV-1-infected adults: results of a randomized controlled trial	Hawken et al., 1997	Another type of study	Randomized clinical trial	QRF
Community-based referral for tuberculosis preventive therapy is effective for treatment completion	Shenoi et al., 2022	Another type of study	Observational evaluation study with comparative analysis	JNBSJ
Short-course isoniazid and pyrazinamide compared with 6-month isoniazid for tuberculosis prevention in HIV-infected adults: The randomized clinical trial	Jasmer et al., 2002	Another type of study	Randomized clinical trial	QRF
Implementation of isoniazid preventive therapy in people living with HIV in Zambia: challenges and lessons	Kagujje et al., 2019	Another type of study	Implementation study	LKAA
Tuberculosis incidence after 36 months' isoniazid prophylaxis in HIV-infected adults in Botswana: a posttrial observational analysis	Samandari et al., 2015	Another type of study	Randomized clinical trial	GCL
Evaluation of the Effect of 3HP vs Periodic 3HP vs 6H in HIV-Positive Individuals	Unidentified author	Another type of study	Study record	QRF
Tuberculosis Preventive Treatment Scale-Up Among Antiretroviral Therapy Patients — 16 Countries Supported by the U.S. President's Emergency Plan for AIDS Relief, 2017–2019	Melgar et al., 2020	Another type of study	Report	JNBSJ
Risk factors for developing active tuberculosis after the treatment of latent tuberculosis in adults infected with human immunodeficiency virus	Amoakwa et al., 2014	Another type of study	Randomized clinical trial	GCL

Preferences of people living with HIV for features of tuberculosis preventive treatment regimens - a discrete choice experiment	Aschmann et al., 2023	Another type of study	Experimental study	GCL
Rifampin and pyrazinamide vs isoniazid for prevention of tuberculosis in HIV-infected persons: an international randomized trial. Terry Beirn Community Programs for Clinical Research on AIDS, the Adult AIDS Clinical Trials Group, the Pan American Health Organization, and the Centers for Disease Control and Prevention Study Group	Gordin et al., 2000	Another type of study	Randomized clinical trial	LKAA
Completion of isoniazid-rifapentine (3HP) for tuberculosis prevention among people living with HIV: Interim analysis of a hybrid type 3 effectiveness-implementation randomized trial	Semitala et al., 2021	Another type of study	Randomized clinical trial	QRF
Annual Tuberculosis Preventive Therapy for Persons With HIV Infection : A Randomized Trial	Churchyard et al., 2021	Another type of study	Randomized clinical trial	LKAA
Effect of isoniazid prophylaxis on mortality and incidence of tuberculosis in children with HIV: randomised controlled trial	Zar et al., 2007	Another type of study	Randomized clinical trial	QRF
Lower TB incidence among PWH after preventive treatment in high-burden MDR TB setting	Sodeke et al., 2023	Conference abstracts	Conference abstracts	JNBSJ
Trends in output of research in clinical prediction models of stroke outcomes: A bibliometric analysis	Mohammed et al., 2021	Conference abstracts	Conference abstracts	JNBSJ
Abstracts of the 37th International Conference on Pharmacoepidemiology & Therapeutic Risk Management, Virtual, August 23, 2021	No authors listed, 2021	Conference abstracts	Conference abstracts	GCL
Oral abstracts of the 11th IAS Conference on HIV Science, 18-21 July 2021	No authors listed, 2021	Conference abstracts	Conference abstracts	GCL
Serum markers and integrative multi-omics of TB diagnosis in advanced HIV	Krishnan et al., 2021	Conference abstracts	Conference abstracts	LKAA
Outcomes of isoniazid preventive therapy among children living with HIV in Kenya	Onyango et al., 2021	Conference abstracts	Conference abstracts	JNBSJ
Effect of isoniazid preventive therapy on prevention of tuberculosis and reduction of all-cause mortality among HIV patients on antiretroviral therapy	Gebremariam et al., 2020	Conference abstracts	Conference abstracts	LKAA
Adjusted analysis of effect of IPT on adverse pregnancy outcomes in women with HIV	Theron et al., 2020	Conference abstracts	Conference abstracts	LKAA
Effectiveness of 3HP annually vs once for HIV-positive people: The WHIP3TB trial	Gebremariam et al., 2020	Conference abstracts	Conference abstracts	JNBSJ
Abstract supplement oral abstracts from the 23rd international AIDS conference, 6-10 july 2020	No authors listed, 2020	Conference abstracts	Conference abstracts	GCL

Evaluation of pulmonary tuberculosis following IPT in Kenyan adults living with HIV	Gersh et al., 2019	Conference abstracts	Conference abstracts	GCL
Oral abstracts of the 22nd International AIDS Conference, 23–27 July 2018, Amsterdam, the Netherlands	No authors listed, 2018	Conference abstracts	Conference abstracts	LKAA
Does the benefit of IPT for persons with HIV who consume alcohol outweigh the risk?	Freiman et al, 2017	Conference abstracts	Conference abstracts	LKAA
AJTMH - Abstract Book	No authors listed, 2016	Conference abstracts	Conference abstracts	JNBSJ
International Congress of Drug Therapy in HIV Infection 23-26 October 2016, Glasgow, UK	No authors listed, 2016	Conference abstracts	Conference abstracts	JNBSJ
8th IAS Conference on HIV Pathogenesis, Treatment & Prevention 19–22 July 2015, Vancouver, Canada	No authors listed, 2015	Conference abstracts	Conference abstracts	QRF
Underutilization of isoniazid drug therapy to prevent TB disease progression in Swaziland	Cronin et al., 2015	Conference abstracts	Conference abstracts	GCL
Track A Basic Science: AIDS2012 Abstract Supplement	No authors listed, 2012	Conference abstracts	Conference abstracts	GCL
Positive predictive value of Interferon-gamma release assay for incident active tuberculosis in HIV-infected persons	Lee et al., 2012	Conference abstracts	Conference abstracts	QRF
Effectiveness of isoniazide prevention therapy in prevention of TB in adult PLHIV under programmatic conditions	Prabhu et al, 2020	Conference abstracts	Conference abstracts	JNBSJ
Modeling the Impact of HIV and TB interventions on South African TB trends: 1990-2019	Kubjane et al., 2020	Conference abstracts	Conference abstracts	GCL
Alcohol use and subotimal adherence to isoniazid in persons with HIV and latet TB	Muyindike et al., 2022	Conference abstracts	Conference abstracts	GCL
Isoniazid preventive therapy among HIV infected patients on antiretroviral therapy diagnosed with latent tuberculosis: A retrospective assessment of the outcome in Tanzania	Marwa et al., 2023	Not available	Not available	GCL
Risk for developing tuberculosis among anergic patients infected with HIV	Moreno et al., 1993	Not available	Not available	JNBSJ
Effect of isoniazid prophylaxis on incidence of active tuberculosis among Thai HIV-infected individuals	Saenghirunvattana, 1996	Not available	Not available	JNBSJ
Brief Report: Yield of Repeat Tuberculin Skin Testing for People Living With HIV in Brazil	Chaisson et al., 2021	Obstinate on the subject	No results associated with TPT	QRF
Routine implementation of isoniazid preventive therapy in HIV-infected patients in seven pilot sites in Zimbabwe	Takarinda et al., 2017	Obstinate on the subject	It does not discuss the development of TB	GCL

Liver Injury Following Isoniazid Preventive Therapy in HIV Patients Attending Halibet National Referral Hospital, Eritrea: A Prospective Cohort Study	Russom et al., 2023	Obstinate on the subject	Unrelated to the topic (focus on liver damage after TPT)	QRF
Determinant factors for the occurrence of tuberculosis after initiation of antiretroviral treatment among adult patients living with HIV at Dessie Referral Hospital, South Wollo, Northeast Ethiopia, 2020. A case-control study	Abdu et al., 2021	Obstinate on the subject	No results associated with TPT	JNBSJ
A prospective cohort study of outcomes for isoniazid prevention therapy: a nested study from a national QI collaborative in Uganda	Sensalire et al., 2020	Obstinate on the subject	Specific focus on adherence and the side effects of using preventive therapy	QRF
Epidemiological survival pattern, risk factors, and estimated time to develop tuberculosis after test and treat strategies declared for children living with human immune deficiency virus	Tsegaye et al., 2023	Obstinate on the subject	Does not address TB risk factors in PLHIV who have undergone TPT	GCL
High incidence of tuberculosis in the first year of antiretroviral therapy in the Botswana National antiretroviral therapy programme between 2011 and 2015	Mupfumi et al., 2019	Obstinate on the subject	No results associated with TPT	LKAA
Isoniazid preventive therapy: Uptake, incidence of tuberculosis and survival among people living with HIV in Bulawayo, Zimbabwe	Nyathi et al., 2019	Obstinate on the subject	All the people who had TPT did not develop TB	QRF
Incidence of tuberculosis among HIV infected individuals on long term antiretroviral therapy in private healthcare sector in Pune, Western India	Dravid et al., 2019	Obstinate on the subject	No results associated with TPT	GCL
High prevalence and incidence of tuberculosis in people living with the HIV in Mandalay, Myanmar, 2011-2017	Phya et al., 2019	Obstinate on the subject	Does not address TB risk factors in PLHIV who have undergone TPT	JNBSJ
Tracking the rate of initiation and retention on isoniazid preventive therapy in a high human immunodeficiency virus and tuberculosis burden setting of Lesotho	Mugomeri et al., 2019	Obstinate on the subject	Does not address TB risk factors in PLHIV who have undergone TPT	LKAA
High Incidence of Tuberculosis Infection in HIV-Exposed Children Exiting an Isoniazid Preventive Therapy Trial	Cranmer et al., 2018	Obstinate on the subject	Does not address TB risk factors in PLHIV who have undergone TPT	QRF
Immunological recovery in tuberculosis/HIV co-infected patients on antiretroviral therapy: implication for tuberculosis preventive therapy	Karo et al., 2017	Obstinate on the subject	No results associated with TPT	QRF

Tuberculosis along the continuum of HIV care in a cohort of adolescents living with HIV in Ethiopia	Jerene et al., 2017	Obstinate on the subject	Does not address TB risk factors in PLHIV who have undergone TPT	LKAA
High Incidence of Tuberculosis in the Absence of Isoniazid and Cotrimoxazole Preventive Therapy in Children Living with HIV in Northern Ethiopia: A Retrospective Follow-Up Study	Alemu, Gebeye, 2016	Obstinate on the subject	Does not address TB risk factors in PLHIV who have undergone TPT	GCL
Tuberculosis prevalence, incidence, and prevention in a South African cohort of children living with HIV	Anyalechi et al., 2022	Obstinate on the subject	Does not address TB risk factors in PLHIV who have undergone TPT	QRF
Implementation and evaluation of an isoniazid preventive therapy pilot program among HIV-infected patients in Vietnam, 2008−2010	Trinh et al., 2015	Obstinate on the subject	Does not document the development of TB in PLHIV who have undergone TPT	GCL
A Clinical Algorithm to Identify HIV Patients at High Risk for Incident Active Tuberculosis: A Prospective 5-Year Cohort Study	Lee et al., 2015	Obstinate on the subject	Does not document the development of TB in PLHIV who have undergone TPT	QRF
Isoniazid preventive therapy use among patients on antiretroviral therapy: a missed opportunity	Kufa et al., 2014	Obstinate on the subject	Does not document the development of TB in PLHIV who have undergone TPT	JNBSJ
Tuberculin sensitivity testing and treatment of latent tuberculosis remains effective for tuberculosis control in human immunodeficiency virus-infected patients in Hong Kong	Lin et al., 2013	Obstinate on the subject	Does not document the development of TB in PLHIV who have undergone TPT	JNBSJ
Completion of isoniazid preventive therapy and survival in HIV-infected, TST-positive adults in Tanzania	Kabali et al., 2011	Obstinate on the subject	Does not document the development of TB in PLHIV who have undergone TPT	GCL
Eligibility for and outcome of treatment of latent tuberculosis infection in a cohort of HIV-infected people in Spain	Diaz et al., 2010	Obstinate on the subject	Does not address TB risk factors in PLHIV who have undergone TPT	JNBSJ
Tuberculosis in asymptomatic HIV-infected adults with abnormal chest radiographs screened for tuberculosis prevention	Agizew et al., 2010	Obstinate on the subject	Does not address TB risk factors in PLHIV	JNBSJ

			who have undergone TPT	
Long-term effectiveness of diagnosing and treating latent tuberculosis infection in a cohort of HIV-infected and at-risk injection drug users.	Golub et al., 2008	Obstinate on the subject	Does not address TB risk factors in PLHIV who have undergone TPT	GCL
HIV-associated tuberculosis in the era of highly active antiretroviral therapy. The Adult/Adolescent Spectrum of HIV Disease Group	Jones et al., 2000	Obstinate on the subject	No results associated with TPT	JNBSJ
Incidence os active tuberculosis among people living with HIV receiving long-term antiretroviral therapy in high TB/HIV burden settings in Thailand: implication for tuberculosis preventive therapy	Suwanpimolkul et al., 2022	Obstinate on the subject	No results associated with TPT	JNBSJ
Latent Tuberculosis among Human Immunodeficiency Virus (HIV) Positive Patients: Prevalence and Correlates	Ajayi et al., 2022	Obstinate on the subject	Does not document the development of TB in PLHIV who have undergone TPT	QRF
Characteristics, Comorbidities, and Outcomes in a Multicenter Registry of Patients With Human Immunodeficiency Virus and Coronavirus Disease 2019	Dandachi et al., 2021	Obstinate on the subject	Unrelated to the topic (focus on COVID)	LKAA
Latent and subclinical tuberculosis in HIV infected patients: a cross-sectional study	Kall et al., 2012	Obstinate on the subject	It only mentions the use of TPT, without documenting its impact on TB	LKAA
Positive rate and risk factors of latent tuberculosis infection among persons living with HIV in Jiangsu Province, China	Zhang et al., 2023	Obstinate on the subject	It only mentions the use of TPT, without documenting its impact on TB	GCL
Determinants of isoniazid preventive therapy completion among people living with HIV attending care and treatment clinics from 2013 to 2017 in Dar es Salaam Region, Tanzania. A cross-sectional analytical study	Robert et al., 2020	Obstinate on the subject	Does not document the development of TB in PLHIV who have undergone TPT	JNBSJ
Strategy to Better Select HIV-Infected Individuals for Latent TB Treatment in BCG-Vaccinated Population	Yang et al., 2013	Obstinate on the subject	Does not address the characteristics of PLHIV who developed TB after TPT	JNBSJ
Tuberculosis preventive treatment uptake among people living with HIV during COVID-19 period in Addis Ababa, Ethiopia: a retrospective data review	Gebreegziabher et al., 2024	Obstinate on the subject	Does not document the development of TB in	LKAA

			PLHIV who have undergone TPT	
Feasibility and efficacy of isoniazid prophylaxis for latent tuberculosis in HIV-infected clients patients in Thailand	Khongphattha nayothin et al., 2012	Obstinate on the subject	All the people who had TPT did not develop TB	JNBSJ
High tuberculosis burden among HIV-infected populations in Thailand due to a low-sensitivity tuberculin skin test	Miyahara et al., 2020	Obstinate on the subject	Does not document the development of TB in PLHIV who have undergone TPT	LKAA
Acceptability and adherence to Isoniazid preventive therapy in HIV-infected patients clinically screened for latent tuberculosis in Dar es Salaam, Tanzania	Shayo et al., 2015	Obstinate on the subject	Does not document the development of TB in PLHIV who have undergone TPT	JNBSJ
Reducing tuberculosis incidence by tuberculin skin testing, preventive treatment, and antiretroviral therapy in an area of low tuberculosis transmission	Elzi et al., 2007	Obstinate on the subject	All the people who had TPT did not develop TB	GCL
Effectiveness of isoniazid treatment for latent tuberculosis infection among human immunodeficiency virus (HIV)-infected and HIV-uninfected injection drug users in methadone programs	Scholten et al., 2003	Obstinate on the subject	Does not address TB risk factors in PLHIV who have undergone TPT	LKAA
HIV patients with latent tuberculosis living in a low-endemic country do not develop active disease during a 2 year follow-up; a Norwegian prospective multicenter study	Pullar et al., 2014	Obstinate on the subject	There was no development of TB in the sample	QRF
Implementation of Isoniazid Preventive Therapy Among HIV-Infected Children at Health Facilities in Nairobi County, Kenya: A Cross-Sectional Study	Mwangi et al., 2019	Obstinate on the subject	It only mentions the use of TPT, without documenting its impact on TB	LKAA
Tolerability of Isoniazid Preventive Therapy in an HIV-Infected Cohort of Paediatric and Adolescent Patients on Antiretroviral Therapy from a Resource-Limited Setting: A Retrospective Cohort Study	Mudzviti et al., 2019	Obstinate on the subject	It does not discuss the development of TB	LKAA
Adherence to tuberculosis preventive therapy among HIV-infected persons in Chiang Rai, Thailand	Ngamvithayap ong et al., 1997	Obstinate on the subject	Does not address TB risk factors in PLHIV who have undergone TPT	GCL
Tuberculosis in HIV programmes in lower-income countries: practices and risk factors	Fenner et al., 2011	Obstinate on the subject	Does not address TB risk factors in PLHIV	LKAA

			who have undergone TPT	
Outcomes of isoniazid preventive therapy among people living with HIV in Kenya: A retrospective study of routine health care data	Karanja et al., 2020	Obstinate on the subject	Focuses on factors associated with IPT completion	LKAA
Factors associated with uptake of isoniazid preventive therapy among children living with HIV in Mwanza region, Tanzania: a cross-sectional study	Tuwa et al., 2024	Obstinate on the subject	It does not discuss the development of TB	QRF
Low prevalence of isoniazid preventive therapy uptake among HIV-infected patients attending tertiary health facility in Lagos, Southwest Nigeria	Busari et al., 2021	Obstinate on the subject	It does not discuss the development of TB	GCL
Coverage of isoniazid preventive therapy among people living with HIV; A retrospective cohort study in Tanzania (2012-2016)	Maokola et al., 2021	Obstinate on the subject	Focuses only on TPT adherence	GCL
Quantiferon-TB Gold: Performance for Ruling out Active Tuberculosis in HIV-Infected Adults with High CD4 Count in Côte d'Ivoire, West Africa	Danel et al., 2014	Obstinate on the subject	Does not address TB risk factors in PLHIV who have undergone TPT	LKAA
Increased uptake of tuberculosis preventive therapy (TPT) among people living with HIV following the 100-days accelerated campaign: A retrospective review of routinely collected data at six urban public health facilities in Uganda	Musaazi et al., 2023	Obstinate on the subject	It does not discuss the development of TB	LKAA
Low level of tuberculosis preventive therapy incompletion among people living with Human Immunodeficiency Virus in eastern Uganda: A retrospective data review	Lwevola et al., 2021	Obstinate on the subject	It does not discuss the development of TB	JNBSJ
A Controlled Trial of Isoniazid in Persons with Anergy and Human Immunodeficiency Virus Infection Who Are at High Risk for Tuberculosis	Gordin et al., 1997	Outro tipo de estudo	Randomized clinical trial	QRF
Effect of isoniazid prophylaxis on incidence of active tuberculosis and progression of HIV infection	Pape et al., 1993	Outro tipo de estudo	Randomized clinical trial	QRF
