# Supplementary material for: Effectiveness of tuberculosis preventive treatment on disease incidence among people living with HIV/AIDS: A systematic review and meta-analysis
Source: PLoS One. 2025 Aug 26;20(8):e0330208. doi: 10.1371/journal.pone.0330208 (PMC12380327; doi:10.1371/journal.pone.0330208)
Supplement: S4 Table — (PDF) [file pone.0330208.s004.pdf]

PLOS

Table S4. Methodological Quality Assessment

Author/Year	Selections	Comparability	Outcomes	Total quality score	Results
Ajema et al, 2024 [18]	***	*	***	7/9	Low risk
Mulatu et al, 2023 [19]	****	*	***	8/9	Low risk
Nyangu et al., 2022 [20]	**	*	***	6/9	Moderate risk
Onyango et al., 2022 [21]	**	*	***	6/9	Moderate risk
Kazibwe et al., 2022 [22]	***	*	***	7/9	Low risk
Geremew et al., 2022 [23]	***	*	**	6/9	Moderate risk
Russom et al., 2022 [24]	****	*	***	8/9	Low risk
Maokola et al., 2021 [25]	***	*	***	7/9	Low risk
Mandalakas et al., 2021 [26]	**	*	***	6/9	Moderate risk
Kebede et al., 2021 [27]	**	*	***	6/9	Moderate risk
Souza et al., 2021 [28]	-	*	**	3/9	High risk
Beshaw, Balcha, Lakew, 2021 [29]	**	*	***	6/9	Moderate risk

Padmapriya et al., 2020 [30]	***	*	***	7/9	Low risk
Aemro, Jember, Anlay, 2020 [31]	***	*	***	7/9	Low risk
Atey et al., 2020 [32]	****	*	**	7/9	Low risk
Mengesha, Ahmed, 2020 [33]	****	*	**	7/9	Low risk
Yirdaw et al., 2019 [34]	**	*	**	5/9	Moderate risk
Sabasaba et al., 2019 [35]	**	*	***	6/9	Moderate risk
Wong et al., 2019 [36]	***	*	***	7/9	Low risk
Ahmed et al., 2018 [37]	***	*	***	7/9	Low risk
Satiavan et al., 2018 [38]	*	*	***	5/9	Moderate risk
Maharaj et al., 2017 [39]	***	*	***	7/9	Low risk
Semu et al., 2017 [40]	****	*	**	7/9	Low risk
Saito et al., 2016 [41]	****	*	***	8/9	Low risk
Ayele, Mourik, Bounten, 2015 [42]	**	*	***	6/9	Moderate risk

Aquino et al., 2015 [43]	***	*	***	7/9	Low risk
Assebe et al., 2015 [44]	****	*	***	8/9	Low risk
Yirdaw et al., 2014 [45]	****	*	***	8/9	Low risk
Masini, Sitienei, Weyeinga, 2013 [46]	***	*	***	7/9	Low risk
Sibanda et al., 2013 [47]	*	*	**	4/9	Moderate risk
Martínez-Pino et al., 2013 [48]	**	*	***	6/9	Moderate risk
Frigati et al., 2011 [49]	**	*	***	6/9	Moderate risk
Golub et al., 2009 [50]	**	*	**	5/9	Moderate risk
Gourevitch et al., 1999 [51]	*	*	**	4/9	Moderate risk
