# Supplementary figures and images for: Effectiveness of tuberculosis preventive treatment on disease incidence among people living with HIV/AIDS: A systematic review and meta-analysis
Source: PLoS One. 2025 Aug 26;20(8):e0330208. doi: 10.1371/journal.pone.0330208 (PMC12380327; doi:10.1371/journal.pone.0330208)

**Fig S1. Geographical distribution of included studies.**

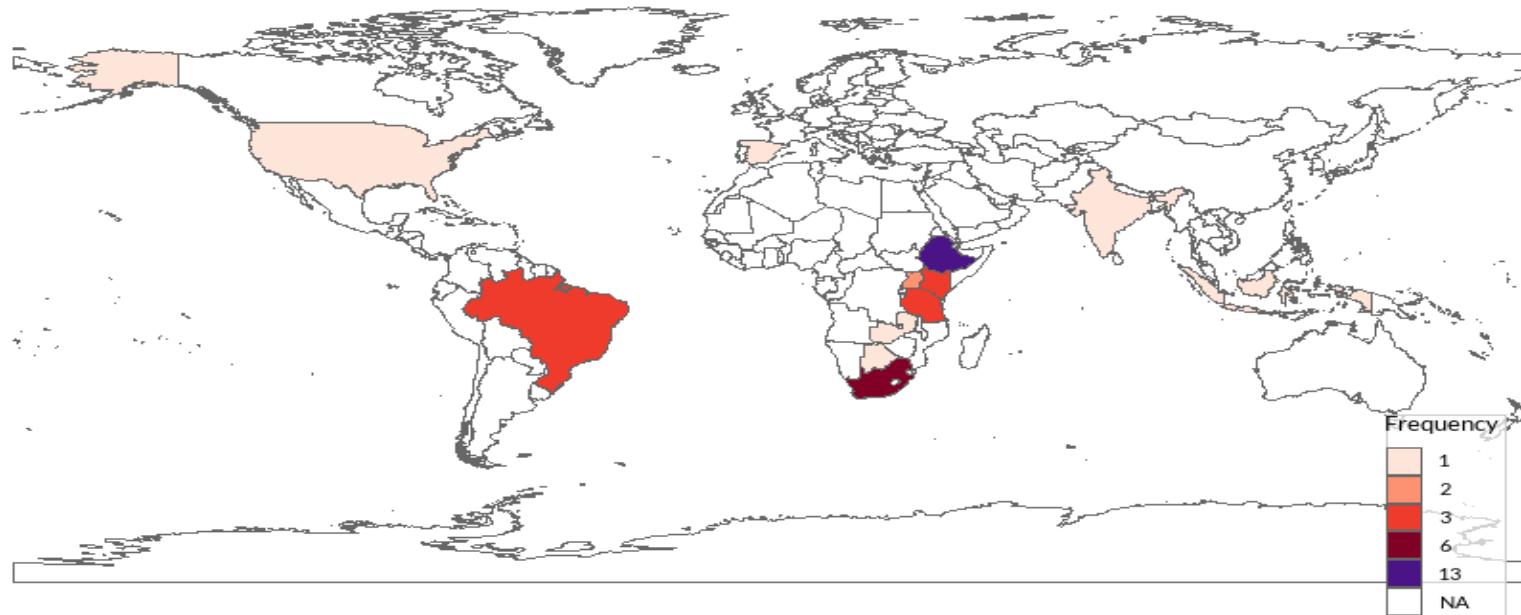

Source: the authors.

Supplement: S1 Fig — (PDF) [file pone.0330208.s005.pdf]
